# AFI-ID: adaptive feature intelligence for intrusion detection

**DOI:** 10.3389/frai.2026.1950536

**Published:** 2026-10-19

**Authors:** S. Antony Joseph Raj, M. Madiajagan

**Affiliations:** School of Computer Science and Engineering, Vellore Institute of Technology, Vellore, Tamil Nadu, India

**Keywords:** Adaptive Feature Intelligence (AFI), attention mechanism, Bidirectional Long Short-Term Memory (BiLSTM), class imbalance, ensemble learning, Extra Trees (ET), extreme gradient boosting (XGB), hybrid feature selection

## Abstract

The increasing sophistication of cyber-attacks necessitates intrusion detection systems (IDS) that are both accurate and computationally efficient, particularly in high-dimensional and class-imbalanced environments. Although ensemble-based IDS models enhance predictive performance, most existing approaches treat feature selection and ensemble aggregation as independent stages, limiting integrated optimization between feature representation and decision-making. To overcome these limitations, this paper proposes Adaptive Feature Intelligence for Intrusion Detection (AFI-ID), an intrusion detection framework that integrates hybrid adaptive feature intelligence with attention-based deep stacking. The proposed AFI module combines statistical relevance measures—Mutual Information and Chi-square with model-based importance derived from XGBoost and Extra Trees through a weighted fusion strategy. This strategy enables the selection of a compact and discriminative feature subset while preserving sensitivity to minority attack classes. At the ensemble level, heterogeneous base learners generate structured meta-features, including log-probability representations, prediction confidence, and entropy. These meta-features are processed by an attention-based Bidirectional Long Short-Term Memory (BiLSTM) meta-classifier, which dynamically weights classifier contributions to achieve context-aware decision fusion. Experimental evaluation on the UNSW-NB15 and CICIDS2017 datasets under binary and multiclass settings demonstrates that AFI-ID achieves strong performance relative to the evaluated baseline classifiers under the experimental protocols considered in this study. AFI-ID achieves 93.31% accuracy with a Receiver Operating Characteristic—Area Under Curve (ROC-AUC) of 0.9849 in binary classification and 79.95% accuracy with a ROC-AUC of 0.9845 in multiclass classification on UNSW-NB15. On CICIDS2017, AFI-ID achieves 98.33% accuracy with a ROC-AUC of 0.9976 in binary classification and 93.54% accuracy with a ROC-AUC of 0.999 in multiclass classification. In repeated UNSW-NB15 binary experiments across five random seeds, AFI-ID achieved a mean accuracy of 93.62%, with paired *t*-tests indicating statistically significant improvements over Logistic Regression (*p* = 1.08 × 10^−10^), Extra Trees (*p* = 1.38 × 10^−5^), and XGBoost (*p* = 3.69 × 10^−5^). These results demonstrate the effectiveness of the proposed adaptive feature selection and attention-based decision fusion strategy under high-dimensional and class-imbalanced network traffic conditions.

## 1 Introduction

The rapid growth of interconnected digital systems has significantly increased the vulnerability of modern networks to sophisticated cyber-attacks. Traditional signature-based intrusion detection methods are increasingly ineffective against dynamic, polymorphic, and previously unseen threats (Ferrag et al., 2020; Maseer et al., 2024). Consequently, Network Intrusion Detection Systems (NIDS) have become essential components of cybersecurity infrastructure, enabling continuous monitoring and identification of anomalous network behavior.

Machine learning (ML) techniques have been widely adopted to enhance intrusion detection by learning complex traffic patterns and distinguishing normal from malicious activities (Pinto et al., 2023; Ahmad et al., 2021). Existing ML-based NIDS architectures commonly employ either individual classifiers or ensemble learning strategies. Although individual classifiers are relatively simple to implement, their performance may be limited by model-specific biases and restricted generalization. Ensemble methods address these limitations by combining heterogeneous learners, with stacking-based architectures providing an effective mechanism for exploiting complementary classifier behavior.

Despite the effectiveness of ensemble-based IDS models, two interrelated limitations remain insufficiently addressed. First, feature selection and ensemble aggregation are commonly designed as separate stages. Conventional feature selection methods typically identify relevant features before classifier training using statistical, filter-based, or model-specific criteria, while the subsequent ensemble stage independently combines classifier outputs. Such decoupling limits the ability of the feature representation to account for the complementary strengths of heterogeneous classifiers. Second, conventional stacking architectures generally rely on fixed meta-classifiers or static aggregation mechanisms that do not explicitly model differences in classifier confidence and prediction uncertainty. These limitations become more pronounced in high-dimensional and class-imbalanced network traffic, where redundant features increase computational complexity and minority attack classes can be inadequately represented.

Recent studies have investigated deep learning and attention mechanisms to improve intrusion detection and ensemble decision fusion. Attention mechanisms can dynamically emphasize informative representations, while LSTM-based architectures provide capabilities for modeling dependencies among sequential or structured inputs. However, these approaches have largely focused either on improving feature representation or on enhancing decision-level fusion, rather than explicitly coupling adaptive feature evaluation with confidence-aware ensemble aggregation.

Our previous work, HAMC-ID, introduced an attention-based meta-classifier for dynamic ensemble fusion, demonstrating the potential of attention mechanisms to improve decision-level aggregation. However, HAMC-ID primarily addressed the classifier-fusion stage and did not incorporate an adaptive feature evaluation mechanism that integrates statistical relevance with model-derived feature importance. Consequently, feature selection and ensemble decision-making remained separate components, leaving an opportunity to strengthen the interaction between the quality of the feature representation and the subsequent ensemble fusion process.

This limitation motivates the research gap addressed in the present study: existing IDS approaches lack an integrated strategy that combines hybrid feature relevance assessment with adaptive, confidence-aware fusion of heterogeneous classifiers. Addressing this gap requires a feature selection mechanism capable of considering complementary statistical and model-based evidence, together with a meta-classifier capable of exploiting structured information about classifier predictions and uncertainty.

To address this gap, this paper proposes Adaptive Feature Intelligence for Intrusion Detection (AFI-ID). AFI-ID employs a hybrid feature intelligence mechanism that combines Mutual Information and Chi-square statistical relevance with feature importance derived from XGBoost and Extra Trees through weighted fusion. The resulting feature subset is designed to retain complementary and discriminative information while reducing feature dimensionality. At the ensemble level, heterogeneous base learners consisting of Logistic Regression, Extra Trees, and XGBoost generate structured meta-features comprising log-probability representations, prediction confidence, and entropy. These meta-features are subsequently processed by an attention-based Bidirectional Long Short-Term Memory (BiLSTM) meta-classifier, which learns to assign context-dependent importance to the available classifier representations.

Thus, unlike approaches that independently perform feature selection and ensemble aggregation, AFI-ID couples adaptive feature evaluation with attention-based decision fusion in a sequentially integrated learning pipeline. This design enables the feature representation to be refined before ensemble learning while allowing the meta-classifier to exploit both classifier predictions and uncertainty information. The effectiveness of the proposed approach is evaluated on the UNSW-NB15 and CICIDS2017 benchmark datasets under binary and multiclass classification settings.

### 1.1 Problem statement

Despite advances in ML-based intrusion detection, existing approaches continue to face challenges associated with high-dimensional feature spaces, class imbalance, and heterogeneous classifier behavior. In particular, feature selection and ensemble aggregation are frequently treated as independent processes, preventing feature relevance from being considered in conjunction with the complementary information provided by different classifiers.

Furthermore, conventional stacking architectures often employ fixed meta-classifiers that primarily operate on classifier predictions without explicitly incorporating prediction confidence and uncertainty. Consequently, the meta-level learner may have limited information for distinguishing reliable predictions from uncertain or conflicting classifier outputs.

Therefore, the research problem addressed in this study is how to integrate hybrid feature relevance assessment with confidence-aware ensemble decision fusion so that redundant feature information is reduced while complementary classifier information is effectively exploited. Specifically, the proposed approach aims to: (i) identify discriminative features using complementary statistical and model-based evidence; (ii) construct informative meta-representations from heterogeneous base classifiers; and (iii) dynamically aggregate classifier information using attention-based learning to improve intrusion detection performance under high-dimensional and imbalanced conditions.

### 1.2 Contributions of the work

The main contributions of this work are summarized as follows:

- Hybrid Adaptive Feature Intelligence: A feature selection mechanism that combines Mutual Information and Chi-square statistical relevance with XGBoost- and Extra Trees-based feature importance through weighted fusion, enabling the identification of a compact and discriminative feature subset.
- Attention-Based Deep Stacking: A heterogeneous stacking architecture based on Logistic Regression, Extra Trees, and XGBoost, coupled with an attention-based BiLSTM meta-classifier for context-aware aggregation of classifier outputs.
- Structured Confidence- and Uncertainty-Aware Meta-Features: A meta-feature representation incorporating log-probability outputs, prediction confidence, and entropy to provide the meta-classifier with complementary information about classifier decisions and prediction uncertainty.
- Comprehensive Evaluation: AFI-ID is evaluated on the UNSW-NB15 and CICIDS2017 datasets under binary and multiclass settings and compared with individual classifiers and ensemble-based baselines to assess its effectiveness, robustness, and generalization.

## 2 Related work

Recent advances in machine learning have significantly improved intrusion detection by addressing the increasing complexity of network traffic. Recent IDS research has explored several complementary directions, including feature selection, ensemble learning, and deep representation learning using recurrent, attention-based, Transformer, and graph neural network architectures. Although these approaches address different aspects of intrusion detection, the interaction between adaptive feature relevance and classifier-level decision fusion remains insufficiently explored. While both approaches have demonstrated effectiveness, they are typically treated as independent stages, limiting integrated optimization between feature representation and decision-making.

This section reviews representative studies in feature selection and ensemble-based intrusion detection, highlighting their contributions and limitations in relation to the proposed AFI-ID framework.

### 2.1 Feature selection techniques for intrusion detection

Feature selection plays a crucial role in intrusion detection due to the high dimensionality and redundancy inherent in modern network traffic datasets. Effective dimensionality reduction not only improves computational efficiency but also enhances model stability by eliminating irrelevant and correlated features.

A significant body of research has focused on statistical filtering methods, which evaluate feature relevance independently of the learning algorithm. For instance, Tripathi et al. (2024) proposed a weighted feature selection framework that prioritizes features based on their impact on the target variable, resulting in reduced feature dimensionality and improved intrusion detection accuracy. Kasongo and Sun (2019) conducted a comparative analysis of classical filtering techniques such as Information Gain and Chi-square, demonstrating measurable performance differences across intrusion detection models. Although these approaches are computationally efficient, their reliance on fixed statistical scores and predefined thresholds limits adaptability under dynamic network traffic conditions. Recent studies have also examined the combined effect of data balancing and feature selection, where techniques such as SMOTE and recursive feature elimination (RFE) enhance detection performance under class-imbalanced conditions (Barkah et al., 2023).

To address the limitations of independent filtering, embedded and model-driven feature selection strategies have been extensively investigated. Ahmed et al. (2025) combined Pearson correlation analysis with Random Forest-based importance estimation to eliminate redundant features in IoT-based intrusion detection systems, achieving improved stability compared to standalone filtering approaches. Similarly, Logeswari et al. (2025a) proposed a dual-layer feature selection mechanism integrated with ensemble classification to enhance detection performance. However, such approaches typically rely on fixed importance weighting or predefined feature subset sizes, which may limit their generalization capability across heterogeneous datasets.

To overcome these challenges, hybrid and advanced feature selection frameworks have been introduced to combine complementary evaluation criteria. Ayad et al. (2024) proposed a hybrid feature selection approach that integrates statistical filtering with optimization-based evaluation to improve anomaly detection performance. Wang J. et al. (2023) introduced a multi-criteria wrapper-based feature selection framework (LVW-MECO) for IoT-based intrusion detection, which incorporates multiple evaluation metrics to enhance detection accuracy. However, the approach relies on search-based optimization and does not explicitly integrate feature selection with ensemble decision-making. Furthermore, Sezgin et al. (2025) investigated multi-objective feature selection using bio-inspired optimization algorithms, highlighting trade-offs between detection accuracy, feature subset size, and computational efficiency, but with high computational cost and limited integration with ensemble learning frameworks. Despite these advancements, existing approaches often rely on computationally intensive search strategies or static selection mechanisms, limiting scalability and adaptability in dynamic intrusion detection environments.

In addition, optimization-based wrapper methods have been extensively explored to identify optimal feature subsets. Nandhini and Santhosh Kumar (2025) employed the Whale Optimization Algorithm (WOA) in conjunction with ensemble classifiers, achieving high detection accuracy through search-based feature refinement. Similarly, Kumari et al. (2024) proposed a heuristic optimization approach for DDoS detection, effectively reducing feature dimensionality. Despite their effectiveness, metaheuristic optimization techniques typically incur high computational cost and may produce unstable feature subsets across different runs, limiting their applicability in real-time intrusion detection systems. Furthermore, feature stability analysis, which evaluates the consistency of selected features across different training scenarios, has received limited attention in intrusion detection research (Ozkan-Okay et al., 2023).

Recent advancements have also explored multi-objective and metaheuristic optimization techniques, such as Gray Wolf Optimization (GWO), to simultaneously optimize feature subset size, classification accuracy, and computational efficiency in high-dimensional environments (Mirjalili et al., 2014). While these approaches provide improved optimization capability, they often introduce additional computational overhead.

Parallel to feature selection advancements, deep learning and ensemble-based frameworks have been investigated to enhance detection robustness. Emirmahmutoğlu and Atay (2025) proposed a feature selection-driven machine learning framework for anomaly detection, while Kaushik et al. (2025) integrated statistical feature selection with machine learning-based IDS models to improve detection performance. Christy et al. (2025) incorporated feature selection within deep learning-based intrusion detection systems to capture complex attack patterns. However, in most existing approaches, feature selection is loosely coupled with downstream ensemble aggregation, limiting integrated optimization between feature representation and classification.

Earlier hybrid filter–wrapper approaches were explored by Doost et al. (2025), who combined statistical filtering with wrapper validation to reduce feature dimensionality. Although effective, these methods typically rely on fixed fusion strategies, limiting adaptability across diverse network conditions.

Overall, existing feature selection techniques have demonstrated significant improvements in intrusion detection performance and dimensionality reduction. However, most approaches rely on static weighting schemes, fixed threshold settings, or computationally intensive optimization processes. Moreover, feature selection is often treated as a preprocessing step, independently of ensemble decision-making. These limitations motivate the need for a unified framework that integrates adaptive feature intelligence with dynamic ensemble learning, as proposed in the AFI-ID model.

Table 1 summarizes representative feature selection–based IDS approaches, highlighting their strategies, classifier integration mechanisms, and limitations. Although these methods demonstrate steady progress in dimensionality reduction and detection performance, several limitations remain. Many approaches rely on fixed weighting schemes, static subset sizes, or heuristic thresholding, which limit adaptability to dynamic network conditions. In addition, feature selection is often weakly coupled with ensemble decision-making, limiting sequential optimization between feature learning and classification. These limitations motivate the need for integrated architecture that combines adaptive feature intelligence with ensemble learning, as proposed in AFI-ID.

**Table 1:** Summary of feature selection–based IDS methods.

Author and references	Feature selection method	Classifier	Key contribution	Limitation
Christy et al. (2025)	Feature selection with deep learning	Deep neural network	Captures complex intrusion patterns	Feature selection not tightly coupled with classifier confidence modeling
Doost et al. (2025)	Filter–wrapper hybrid FS	Ensemble classifier	Effective dimensionality reduction	Fixed fusion strategy; lacks adaptive feature–ensemble interaction
Kasongo and Sun (2019)	Traditional filter FS	ML classifiers	Comparative evaluation of filtering techniques	No hybrid fusion or adaptive interaction with classifiers
Tripathi et al. (2024)	Weighted feature selection	ML classifiers	Improves detection accuracy and reduces feature space dimensionality	Static weighting; lacks adaptive feature importance learning
Nandhini and Santhosh Kumar (2025)	Metaheuristic FS (WOA)	Ensemble classifiers	High detection accuracy via search-based optimization	High computational cost; limited scalability
Kaushik et al. (2025)	Hybrid FS with ML Models	ML classifiers	Improves detection performance using statistical FS	Limited integration with ensemble decision dynamics
Logeswari et al. (2025a)	Dual-layer hybrid FS	Ensemble classifiers	Improves feature selection effectiveness and classification performance through layered FS	Fixed thresholds; lacks adaptive feature weighting
Kumari et al. (2024)	Metaheuristic FS (heuristic optimization)	ML classifiers	Reduces dimensionality for DDoS detection	Non-deterministic feature selection; slow convergence
Ahmed et al. (2025)	Pearson + RF (hybrid FS)	Random Forest	Reduces redundancy in IoT IDS	Fixed feature subset; lacks dynamic optimization
Ayad et al. (2024)	Hybrid feature selection	ML classifiers	Combines multiple selection strategies	Manual tuning of feature subset size
Emirmahmutoğlu and Atay (2025)	Feature selection-driven framework	ML classifiers	Improves anomaly detection performance	Limited integration with ensemble adaptation
Barkah et al. (2023)	RFE feature selection	ML classifiers	Improves performance under class imbalance using SMOTE and RFE	Static feature selection; not jointly optimized with model learning or ensemble aggregation
Ozkan-Okay et al. (2023)	Feature selection evaluation	ML classifiers	Evaluates feature robustness and stability	Lacks adaptive feature intelligence
AFI-ID (proposed)	Hybrid adaptive feature intelligence	LR + ET + XGB + BiLSTM	Adaptive fusion of statistical and model-based importance; integrated with attention-based ensemble learning	Slightly higher computational cost than pure filter methods

### 2.2 Ensemble and stacking-based intrusion detection models

Ensemble learning has been widely adopted in intrusion detection to overcome the limitations of single classifiers, particularly in heterogeneous and high-volume network environments. By combining multiple base learners, ensemble models improve predictive stability and reduce model-specific bias. Stacking-based architectures further enhance performance by introducing a meta-classifier to learn higher-level decision boundaries. Ensemble methods improve generalization by reducing variance and expanding the hypothesis space (Dietterich, 2000). In intrusion detection systems, heterogeneous classifier combinations with voting-based aggregation have demonstrated improved performance on benchmark datasets (Ahmad et al., 2021; Bibers et al., 2024). However, most aggregation mechanisms are predefined and cannot dynamically adjust the contribution of individual base learners under changing traffic conditions, limiting their ability to effectively capture evolving intrusion patterns. Stacking-based architectures address this limitation by introducing a meta-classifier that learns higher-level decision boundaries from base classifier outputs.

Tree-based stacking models, including Decision Trees, Random Forests, and gradient boosting frameworks, have demonstrated strong performance on benchmark datasets such as NSL-KDD and UNSW-NB15 (Farha and Ahmed, 2024). Similarly, deep stacking assemblies, which incorporate the architecture of neural networks, are reported to be more resilient when implemented in Internet-of-Things (IoT) and cloud-based intrusion detection environments (Alalwany et al., 2025). However, many existing stacking architectures use fixed meta-learners and invariant feature representations, limiting adaptability to changing traffic distributions and heterogeneous attack patterns.

Mechanisms of adaptive and representation-aware ensemble learning have also been explored. Attention-enhanced frameworks, such as BiLSTM-based intrusion detection architectures, have demonstrated effectiveness in capturing complex patterns and improving detection of low-frequency attacks (Singh et al., 2025). Antony Joseph Raj and Madiajagan proposed HAMC-ID, an attention-based stacking framework that employs a BiLSTM meta-classifier to dynamically weight heterogeneous base classifiers (Antony Joseph Raj and Madiajagan, 2025). Although HAMC-ID improves decision-level aggregation, it does not incorporate hybrid adaptive feature selection or explicitly model feature-level importance. As a result, feature representation and ensemble aggregation remain decoupled, limiting integrated optimization.

The proposed AFI-ID framework addresses this limitation by integrating hybrid adaptive feature intelligence with attention-based stacking, enabling integrated feature–decision learning within a unified architecture. In contrast to prior approaches, AFI-ID incorporates structured meta-feature representations, including prediction confidence, entropy-based uncertainty, and log-probability representations, to enhance context-aware aggregation.

Similarly, online ensemble methods such as Kitsune utilize ensembles of autoencoders for lightweight real-time intrusion detection (Mirsky et al., 2018). Although these approaches improve adaptability or efficiency, they do not jointly optimize feature refinement and ensemble aggregation within a single framework. Modern heterogeneous stacking approaches emphasize classifier diversity to improve robustness and reduce false positives. However, systematic meta-feature modeling remains limited in existing IDS frameworks. In particular, predictive confidence, entropy-based uncertainty, and inter-classifier relationships are rarely incorporated into stacking-based aggregation strategies. These models typically treat base classifier outputs as fixed inputs to the meta-learner, thereby neglecting the modeling of contextual confidence, temporal relationships, and strategy-aware weighting mechanisms.

Although ensemble and stacking-based IDS models have demonstrated strong performance, feature selection and ensemble aggregation are not consistently integrated within the same learning pipeline across existing approaches. In addition, limited attention has been given to structured meta-feature modeling and confidence-aware aggregation. These limitations motivate the development of AFI-ID, which integrates hybrid adaptive feature intelligence with attention-guided stacking within a unified framework.

Unlike recent attention-based ensemble intrusion detection frameworks that primarily focus on adaptive classifier aggregation, AFI-ID establishes a coordinated sequential integration of adaptive feature intelligence and attention-guided ensemble learning within an integrated learning pipeline. Existing attention-based IDS models, including BiLSTM-based stacking approaches, typically operate on features obtained through conventional preprocessing or fixed feature selection procedures. In contrast, AFI-ID introduces a hybrid adaptive feature intelligence mechanism that combines statistical relevance and model-based importance through weighted fusion and adaptive threshold refinement prior to ensemble learning. Furthermore, AFI-ID employs structured meta-feature representations incorporating prediction confidence, entropy-based uncertainty, and log-probability information, enabling more context-aware aggregation than conventional probability-based stacking architectures. This coordinated interaction between feature refinement and attention-guided decision fusion constitutes the primary technical distinction of AFI-ID.

### 2.3 Transformer-based intrusion detection

Transformer architectures have emerged as an important direction in recent intrusion detection research because self-attention enables contextual relationships among traffic representations to be modeled. Mercha et al. (2023) proposed Trans-IDS, a Transformer-based intrusion detection system evaluated on UNSW-NB15 and NSL-KDD. Unlike approaches that explicitly apply feature-selection algorithms, Trans-IDS does not rely on conventional feature selection and instead learns contextualized representations for categorical and numerical features.

Long et al. (2024) developed a Transformer-based NIDS for cloud security, using attention mechanisms to model relationships between input features and intrusion types. The study demonstrates the applicability of Transformer architectures to cloud-oriented intrusion detection. Xi et al. (2024) subsequently proposed IDS-MTran, which combines multi-scale convolutional feature extraction, Patching with Pooling, multi-scale Transformer blocks, and Cross Feature Enrichment. The model was evaluated on NSL-KDD, CIC-DDoS2019, and UNSW-NB15 and was designed to capture intrusion patterns at multiple feature scales.

More recent work has continued to extend Transformer-based IDS architectures. Abdelnaby et al. (2026) proposed a Transformer-based NID framework for fog computing and evaluated Transformer variants on NSL-KDD and IoT-20, including binary and multiclass classification. Martin-Reizabal et al. (2026) investigated flow-to-window aggregation, treating network flows as tokens within temporal windows and using self-attention to model relationships among concurrent flows.

These studies demonstrate the effectiveness of Transformer-based contextual representation learning. AFI-ID addresses a different design objective: rather than replacing explicit feature evaluation with learned contextual representations, it combines statistical relevance measures with model-based feature importance before heterogeneous ensemble learning. The subsequent attention-based BiLSTM operates on structured classifier-level meta-features, including log-probability, confidence, and entropy representations.

### 2.4 Graph neural network-based intrusion detection

Graph neural networks provide another recent direction for intrusion detection by representing relationships among network entities, flows, or hosts. Sun et al. (2024) proposed GNN-IDS, which uses graph representations to model network connectivity and applies GNN inference to intrusion detection. The study further incorporates an attack graph to support analysis of malicious actions.

Tran and Park (2024) proposed FN-GNN, a graph-embedding approach for network intrusion detection that constructs graph representations from network-flow information and employs GCN and SAGEConv components. The approach was evaluated using CICIDS2017 and UNSW-NB15.

Recent work has also investigated hybrid graph and attention architectures. Xie and Liu (2026) proposed a GNN–Transformer intrusion detection model that combines graph-based structural representation with Transformer-based contextual modeling through dual-domain feature fusion.

These studies highlight the value of relational representations for intrusion detection. AFI-ID adopts a different representation paradigm: it operates on flow-level tabular features and does not require explicit graph construction. Its contribution instead focuses on hybrid feature relevance assessment and attention-based classifier-level fusion, thereby complementing graph-based IDS approaches rather than directly competing with their graph representation mechanism.

### 2.5 Attention-based and hybrid deep learning IDS

Attention mechanisms have also been increasingly incorporated into IDS architectures to emphasize informative spatial, temporal, or feature-level representations. Zhang et al. (2023) proposed a BiLSTM model with a multi-head attention mechanism for network intrusion detection. More recent work has combined attention with convolutional and recurrent architectures. A dual-attention CNN-BiLSTM model uses separate attention mechanisms to emphasize important local and temporal information in network traffic (Zhang et al., 2025).

Attention has also been integrated with ensemble learning. Singh et al. (2025) proposed an attentional LSTM-ensemble architecture in which an attention-based LSTM performs temporal-saliency feature extraction followed by an ensemble of gradient-boosting classifiers. The study also investigates imbalance mitigation through SMOTE and focal loss.

These studies demonstrate that attention can improve representation learning and, in some cases, can be combined with ensemble classifiers. AFI-ID differs in that attention is applied at the meta-classification stage after heterogeneous base learners have generated structured meta-features. In particular, AFI-ID combines log-probability representations, prediction confidence, and entropy-based uncertainty with an attention-based BiLSTM to support context-aware classifier aggregation.

### 2.6 Positioning of AFI-ID

Recent IDS research therefore encompasses several complementary directions. Transformer-based approaches emphasize contextual representation learning, GNN-based methods model relational dependencies among network entities, and attention-based CNN/LSTM architectures emphasize informative feature or temporal representations. Ensemble approaches further exploit classifier diversity, while feature-selection studies seek to reduce redundant and irrelevant input dimensions.

AFI-ID is positioned at the intersection of these feature- and decision-level considerations. It does not attempt to replace Transformer or GNN representation learning. Instead, it addresses the specific problem of integrating hybrid feature relevance assessment with confidence-aware ensemble decision fusion. The AFI module combines Mutual Information and Chi-square with model-derived importance from XGBoost and Extra Trees, while the ensemble stage uses Logistic Regression, Extra Trees, and XGBoost to generate structured log-probability, confidence, and entropy meta-features. These representations are subsequently processed by an attention-based BiLSTM meta-classifier.

Accordingly, the principal distinction of AFI-ID is the coupling of hybrid statistical/model-based feature evaluation with attention-based meta-level decision fusion. This differentiates the proposed framework from Transformer-only representation learning, graph-based relational modeling, and attention-based feature extraction approaches while providing a complementary approach to heterogeneous ensemble intrusion detection. Table 2 summarizes representative recent IDS approaches and highlights their primary methodological mechanisms in relation to the proposed AFI-ID framework.

**Table 2:** Positioning of recent intrusion detection approaches and AFI-ID.

Approach	Representative studies	Primary mechanism	AFI-ID distinction
Transformer-based IDS	Mercha et al., 2023; Long et al., 2024; Xi et al., 2024; Abdelnaby et al., 2026; Martin-Reizabal et al., 2026	Contextual/self-attention representation learning	Explicit hybrid feature relevance + meta-level fusion
GNN-based IDS	Sun et al., 2024; Tran and Park, 2024	Relational/graph representation learning	Flow-level tabular representation without graph construction
GNN–Transformer	Xie and Liu, 2026	Graph + contextual representation fusion	Feature-selection + classifier-level attention fusion
Attention CNN/BiLSTM	Zhang et al., 2023, 2025	Feature/temporal attention	Attention applied to classifier meta-features
Attention-based ensemble	Singh et al., 2025	Attention-LSTM + boosting ensemble	Adds confidence, entropy and log-probability meta-features
Hybrid feature selection	Wang J. et al., 2023; Ahmed et al., 2025; Logeswari et al., 2025a,b; Ayad et al., 2024; Barkah et al., 2023; Sezgin et al., 2025; Ozkan-Okay et al., 2023; Emirmahmutoğlu and Atay, 2025; Kaushik et al., 2025; Christy et al., 2025; Doost et al., 2025	Statistical/model/optimization-based feature selection	Explicit weighted fusion of statistical and model-based importance
AFI-ID (proposed)	–	MI + Chi-square + XGB/ET + LR/ET/XGB + attention-BiLSTM	Integrated adaptive feature intelligence and confidence-aware meta-fusion

As summarized in Table 2, recent IDS research spans contextual representation learning with Transformers, relational representation learning with GNNs, attention-based spatial and temporal modeling, and hybrid feature-selection strategies. AFI-ID differs by combining hybrid statistical and model-based feature evaluation with confidence- and uncertainty-aware attention-based meta-level fusion.

## 3 Proposed AFI-ID framework

To address the limitations of modern intrusion detection systems, this work proposes AFI-ID, an integrated architecture that combines hybrid adaptive feature intelligence with an attention-based deep-stacking ensemble. Unlike conventional pipelines that treat feature selection and ensemble aggregation as unrelated stages, AFI-ID integrates adaptive feature selection with attention-based ensemble aggregation within a coordinated learning pipeline. The feature-selection and ensemble-learning components are optimized sequentially, while their outputs are explicitly connected through the selected feature representation and structured meta-features. This sequential integration establishes a direct methodological connection between feature representation refinement and subsequent classifier decision-making.

Figure 1 illustrates the overall architecture of the proposed AFI-ID framework. The pipeline begins with raw network traffic data, which is divided into training and testing sets. The training data undergoes preprocessing, including cleaning, encoding, scaling, and class balancing, to ensure data consistency and quality.

**Figure 1:**
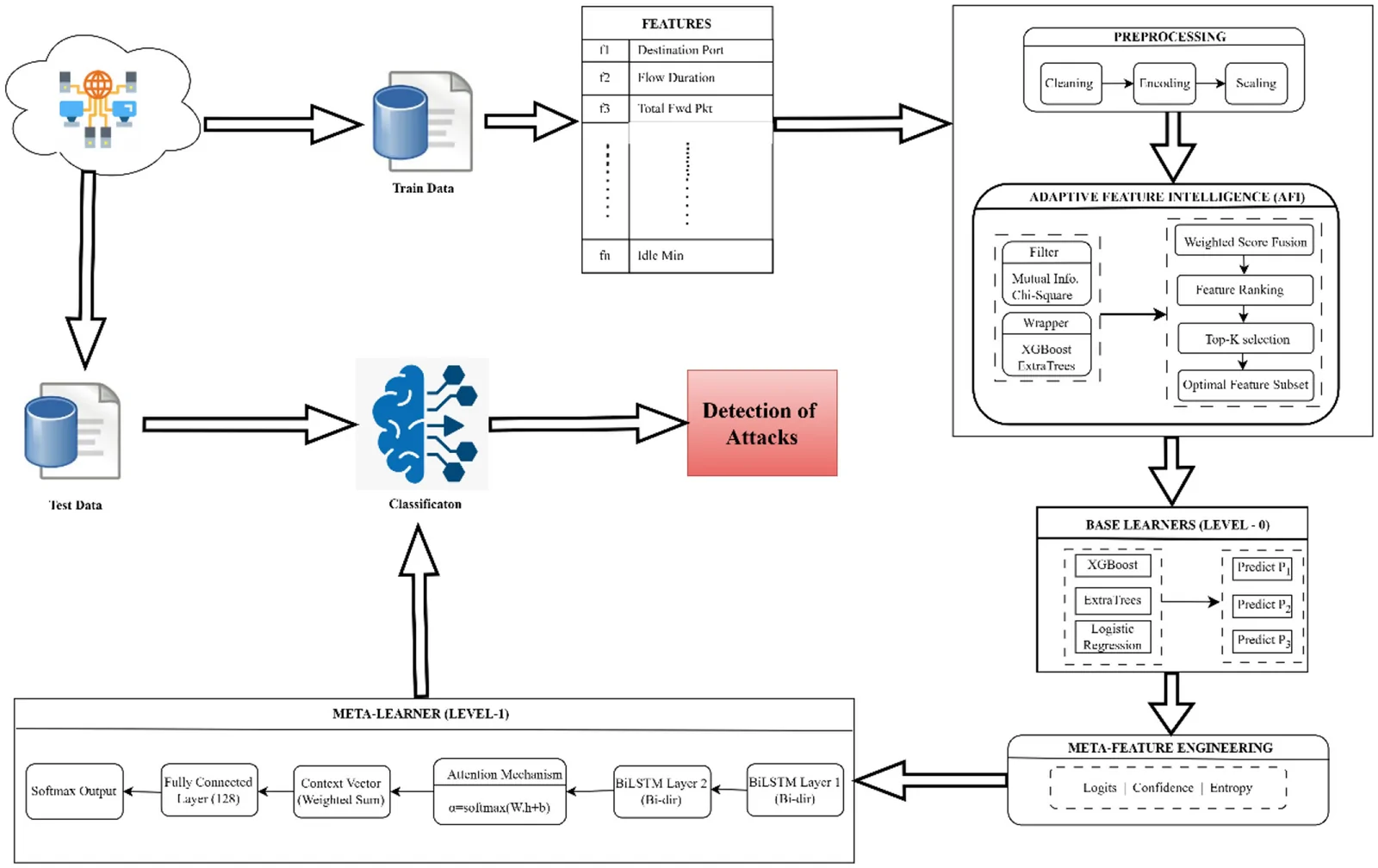
AFI-ID framework architecture.

The processed data is then passed to the AFI module, where statistical filtering methods (MI and Chi-square) are combined with model-based predictive importance derived from XGB and ET through a weighted fusion strategy to select an optimal subset of discriminative features.

These selected features are fed into heterogeneous base learners, including Logistic Regression, ET, and XGB, which generate prediction outputs. Structured meta-features such as log-probability representations, prediction confidence, and entropy-based uncertainty are then extracted from these outputs.

Although the classifier sequence contains only three elements, the sequence represents heterogeneous decision representations generated by LR, ET, and XGB rather than conventional long temporal observations. The BiLSTM is therefore used as a compact sequence model to capture contextual dependencies among the classifier-specific representations, while the attention mechanism adaptively weights the resulting representations. The choice is motivated by the structured nature of the meta-features rather than by long-range temporal dependencies. To assess whether this recurrent formulation provides a meaningful advantage over simpler alternatives, the proposed BiLSTM with attention is compared experimentally with an MLP and an attention-pooling model in Section 6.

### 3.1 Data pre-processing

The performance of intrusion detection systems is highly dependent on the quality and consistency of input data. The UNSW-NB15 and CICIDS2017 datasets contain heterogeneous attributes, including numerical flow-based statistics and categorical protocol-related features. To ensure numerical stability, reduce noise, and improve model learning efficiency, a structured preprocessing pipeline is adopted, as illustrated in Figure 2.

**Figure 2:**
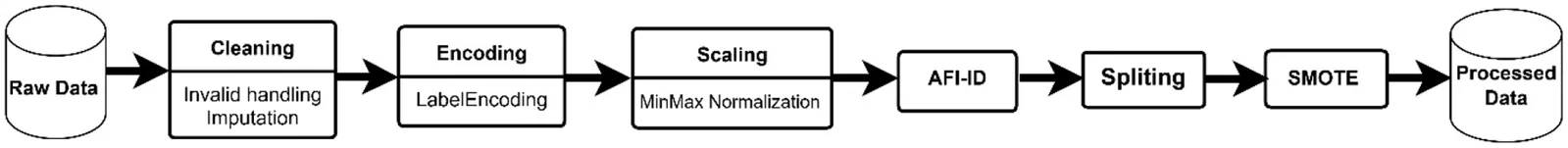
Detailed data preprocessing pipeline of the AFI-ID.

The preprocessing stage begins with data cleaning, where irrelevant identifiers and non-informative attributes are removed. Invalid entries are treated as missing values, and numerical inconsistencies are corrected through appropriate type conversion. Missing values are handled using median imputation to preserve dataset size while reducing the influence of extreme values.

Next, categorical encoding is applied to protocol-related attributes such as *proto, service*, and *state*. These categorical variables are transformed into numerical representations using label encoding. Given a categorical label set:


$$\begin{array}{l} L=\left\{l_{1},l_{2},\ldots ,l_{n}\right\} \end{array}$$

each label is mapped to an integer value as:


$$\begin{array}{l} f\left(l_{i}\right)=i-1,\forall i\in \left[1,n\right] \end{array}$$

This transformation ensures compatibility with machine learning models while avoiding the dimensional expansion associated with one-hot encoding. For binary classification, labels are encoded as 0 (normal) and 1 (intrusion), whereas in the multiclass setting, each attack category is assigned a unique numerical identifier.

To address variations in feature scale, Min–Max normalization is applied. Each feature value x is rescaled to the range [0, 1] using Equation 1:


$$\begin{array}{l} x^{\prime }=\frac{x-\min\left(x\right)}{\max\left(x\right)-\min\left(x\right)} \end{array}$$ (1)

where *x*′ denotes the normalized feature value. This transformation reduces the dominance of large-magnitude features and improves convergence stability during training.

The dataset is then partitioned into training, validation, and testing subsets to ensure reliable performance evaluation. To address class imbalance, Synthetic Minority Over-sampling Technique (SMOTE) (Chawla et al., 2002) is applied exclusively to the training data. This generates synthetic samples for minority classes, reducing bias toward majority classes while preserving the integrity of the evaluation process. The test set remains completely isolated to prevent data leakage.

The class distributions before and after applying SMOTE for the UNSW-NB15 and CICIDS2017 training data are illustrated in Figures 3, 4, respectively. Figure 4 represents the CICIDS2017 experimental training subset used for the SMOTE procedure rather than the complete cleaned dataset. The figure therefore illustrates the class-balancing procedure applied to the training data and should not be interpreted as the class distribution of the complete CICIDS2017 dataset.

**Figure 3:**
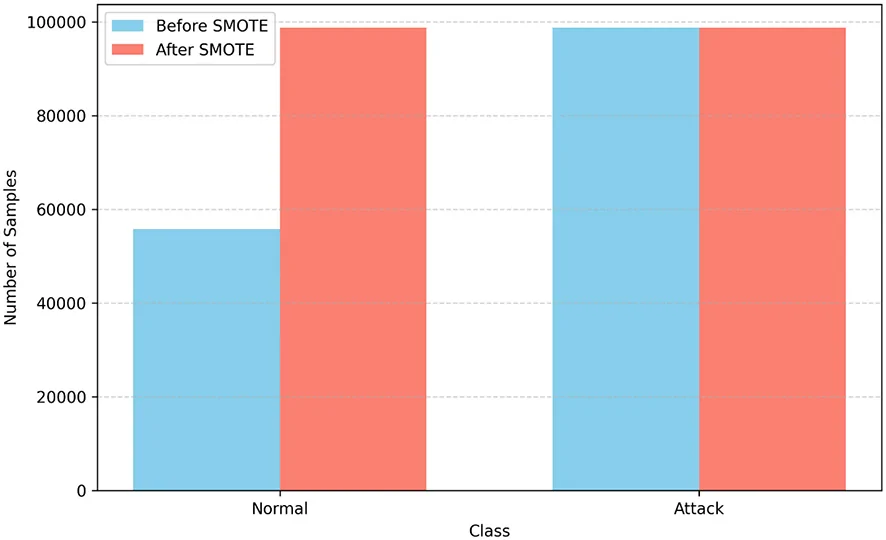
Class distribution UNSW-NB15 before and after SMOTE.

**Figure 4:**
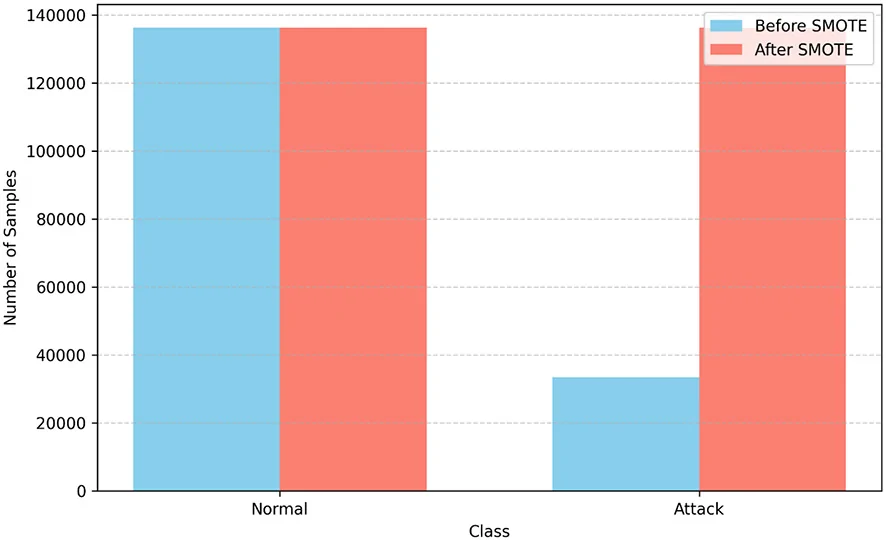
Class distribution of the CICIDS2017 experimental training subset before and after SMOTE.

After preprocessing, the refined dataset is forwarded to the AFI-ID module, where hybrid feature scoring and selection are performed.

### 3.2 Mathematical formulation of hybrid adaptive feature intelligence

High-dimensional intrusion detection data is often characterized by redundant or non-informative features. To address the limitations of single-criterion feature evaluation, AFI-ID employs a hybrid adaptive feature intelligence mechanism that integrates statistical dependency analysis with model-driven importance estimation. Let the dataset be represented as *D* = {*X, Y*}, where *X* denotes the feature space and *Y* represents the class labels.

#### 3.2.1 Filter-based statistical scoring

Two complementary statistical measures are used to quantify the dependency between individual features and the target variable: Mutual Information (MI) and the Chi-square (χ^2^) statistic. Mutual Information is used to quantify the dependency between each feature and the class label, as defined in Equation 2:


$$\begin{array}{l} MI\left(X_{i},\;Y\right)=\underset{x_{i}}{\sum }\underset{y}{\sum }p\left(x_{i},\;y\right)\;\log\left(\frac{p\left(x_{i},\;y\right)}{p\left(x_{i}\right)\;p\left(y\right)}\right) \end{array}$$ (2)

where *p*(*x*_*i*_, *y*) refers to the joint probability distribution, *p*(*x*_*i*_), and *p*(*y*) are the marginal probabilities. A larger mutual information value indicates stronger dependency between the feature and the label of the class. The chi-square statistic is used to determine the difference between the observed and expected frequencies, as defined in Equation 3:


$$\begin{array}{l} \chi^{2}\left(X_{i},\;Y\right)=\;\underset{j}{\sum }\frac{{\left(O\;-\;E\right)}^{2}}{E} \end{array}$$ (3)

where *O*_*j*_ and *E*_*j*_ denote the observed and expected frequencies respectively. Higher values indicate stronger statistical association.

To ensure scale consistency between heterogeneous statistical measures, the filter score is computed after applying min–max normalization to both *MI* and χ^2^ values, since they operate on different numerical scales:


$$\begin{array}{l} {\tilde{M}I}_{i}=\frac{MI_{i}-\;min\left(MI\right)}{\max\left(MI\right)-\;min\left(MI\right)}\; \end{array}$$

$$\begin{array}{l} {\tilde{\chi }}_{i}^{2}=\frac{\chi_{i}^{2}-\;min\left(\chi^{2}\right)}{\max\left(\chi^{2}\right)-\;min\left(\chi^{2}\right)}\; \end{array}$$

The normalized filter score is then computed as defined in Equation 4:


$$\begin{array}{l} S_{i}^{filter}=\frac{{\tilde{M}I}_{i}+{\tilde{\chi }}_{i}^{2}}{2} \end{array}$$ (4)

This normalization ensures scale consistency and prevents dominance of any single statistical measure, resulting in a more balanced hybrid feature evaluation. All normalization operations are performed across the feature set prior to ranking.

#### 3.2.2 Model-based predictive importance

Whereas, the filter methodologies measure statistical associations independently of the process of training models, Model-based feature-importance methods estimate the predictive relevance of features from the learned parameters or structural properties of trained models. To estimate feature importance during training, AFI-ID uses two heterogeneous ensemble learners, including XGB and ET.

Let $Impx_{i}^{XGB}$ and $Imp_{i}^{ET}$ denote the normalized importance [scaled to [0, 1] using min–max normalization] of feature *X*_*i*_ obtained from XGB and ET, respectively. The model-based predictive importance score is calculated as defined in Equation 5:


$$\begin{array}{l} S_{i}^{model}=\frac{{I\tilde{m}p}_{i}^{XGB}+{I\tilde{m}p}_{i}^{ET}}{2} \end{array}$$ (5)

The use of two heterogeneous ensemble learners reduces the bias associated with a single embedded model and increases the strength of importance estimation on a wide range of datasets.

#### 3.2.3 Hybrid weighted fusion strategy

AFI-ID employs a weighted fusion strategy:


$$\begin{array}{l} S_{i}=\alpha S_{i}^{filter}+\beta S_{i}^{wrapper} \end{array}$$ (6)

subject to α + β = 1.

Based on validation-based sensitivity analysis, α = 0.2 and β = 0.8 were selected as the final fusion coefficients. The selection prioritized validation Macro-F1 because of the class-imbalanced nature of intrusion detection, while validation accuracy and Receiver Operating Characteristic—Area Under Curve (ROC-AUC) were considered secondary performance indicators. Under the α-β sensitivity experiment, the configuration α = 0.2 and β = 0.8 achieved a validation Macro-F1 of 0.9252 and ROC-AUC of 0.9870.

The proposed hybrid scoring function in Equation 6 performs a linear scalarization of two feature-evaluation criteria: statistical relevance and predictive importance. The resulting scalar score provides a computationally efficient mechanism for ranking features without requiring a Pareto-based multi-objective optimization procedure. To ensure comparability among the heterogeneous feature-selection criteria, the MI, χ^2^, XGB, and ET importance values are independently normalized to the [0, 1] range using min–max normalization before fusion. The normalized MI and χ^2^ scores are averaged to obtain the statistical filter score, while the normalized XGB and ET importance scores are averaged to obtain the model-based predictive importance score. The final hybrid score is then obtained by weighting these two normalized components using α = 0.2 and β = 0.8 and is subsequently normalized to the [0, 1] range for feature ranking and threshold-based selection. Therefore, the importance values reported in Table 3 and visualized in Figure 5 are normalized hybrid feature-importance scores, rather than raw MI, χ^2^, XGB, or ET importance values.

**Table 3:** Hyperparameter settings of the proposed AFI-ID framework.

Component	Parameter	Value	Description
Feature selection	Candidate thresholds T	0.10, 0.15, 0.20, 0.25, 0.30, 0.35, 0.40, 0.45, 0.50	Validation-based threshold search
Feature selection	Selection rule		Retain features with hybrid scores greater than or equal to the optimal threshold
Feature selection	Threshold selection criterion	Validation Macro-F1	Criterion used to select τ^*^
Hybrid feature fusion	α	0.2	Weight for statistical feature score
Hybrid feature fusion	β	0.8	Weight for model-based feature score
Feature selection	Optimal threshold τ^*^ UNSW-NB15	0.10	Selected using validation Macro-F1 on UNSW-NB15
Feature selection	Final selected features UNSW-NB15	17	Corresponding to τ^*^= 0.10 on UNSW-NB15

**Figure 5:**
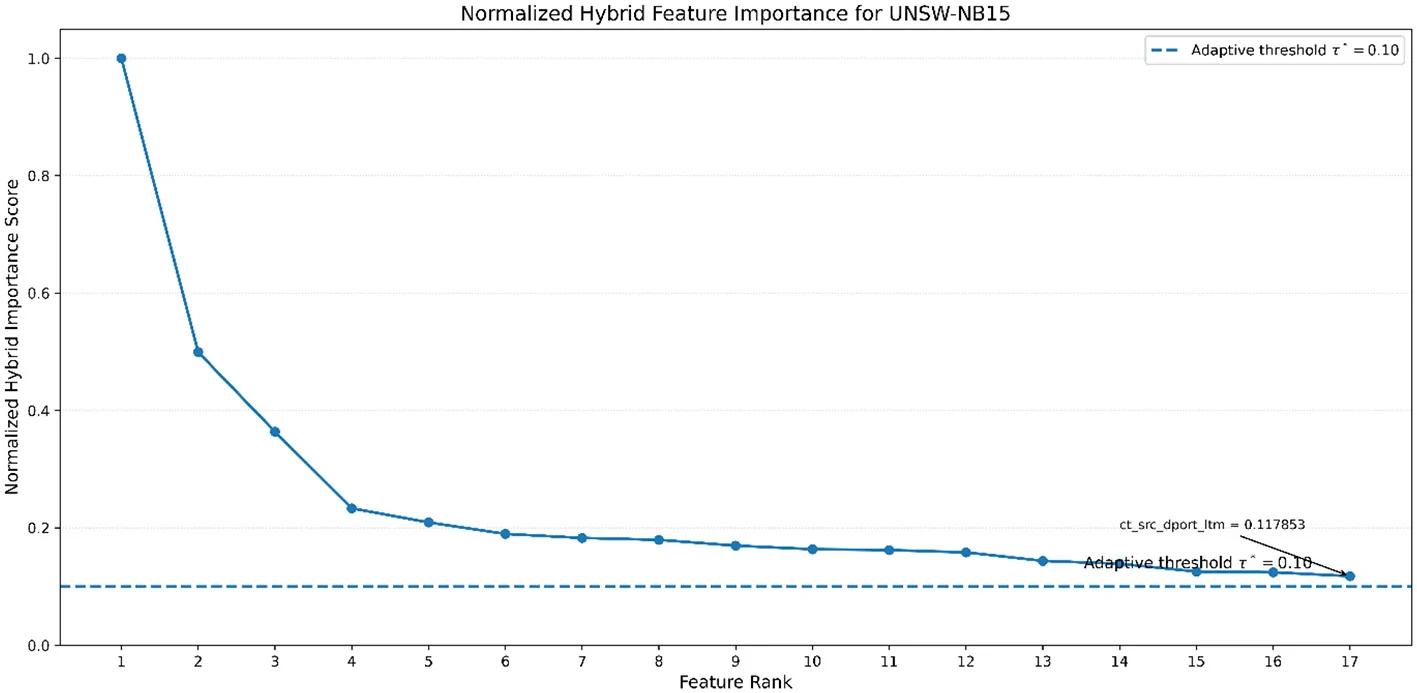
Normalized hybrid feature-importance distribution for UNSW-NB15.

Unlike Pareto-based multi-objective optimization approaches, which require maintaining a set of non-dominated solutions, the proposed scalarization enables efficient computation and direct feature ranking. This is particularly important for large-scale intrusion detection datasets, where computational efficiency and scalability are critical.

Thus, the AFI-ID framework combines two complementary feature-evaluation criteria—statistical relevance and predictive importance—through a computationally tractable weighted feature-ranking procedure suitable for large-scale intrusion detection. The adaptivity of AFI-ID arises from the data-dependent hybrid feature scores and the validation-based selection of the feature-retention threshold. Because the threshold is selected according to the validation performance of the resulting feature subset rather than being imposed as a fixed feature count, the number of retained features can vary according to the distribution of hybrid feature scores, while α and β remain fixed empirical fusion coefficients after validation-based sensitivity analysis.

#### 3.2.4 α-β sensitivity analysis

To examine the sensitivity of the hybrid feature-fusion mechanism to the weighting coefficients, an additional validation-based sensitivity analysis was conducted. The coefficients α and β were constrained such that α + β = 1, where α controls the contribution of the normalized statistical filter score and β controls the contribution of the normalized model-based predictive importance score. The candidate configurations included 11 α-β combinations satisfying α + β = 1, namely (0.0, 1.0), (0.1, 0.9), (0.2, 0.8), (0.3, 0.7), (0.4, 0.6), (0.5, 0.5), (0.6, 0.4), (0.7, 0.3), (0.8, 0.2), (0.9, 0.1), and (1.0, 0.0). For each configuration, the hybrid feature scores were computed using the training partition only, the adaptive threshold was determined using the validation partition, and the resulting feature subset was evaluated using the corresponding validation performance. The held-out test set was not used during weight selection. The validation results for the evaluated α-β configurations are presented in Table 4.

**Table 4:** Validation-based sensitivity analysis of the α-β fusion coefficients in AFI-ID.

α	β	Selected features	Validation accuracy (%)	Validation Macro-F1	Validation ROC-AUC
0.2	0.8	21	92.9524	0.9252	0.9870
0.1	0.9	21	92.9659	0.9250	0.9868
0.5	0.5	21	92.7952	0.9233	0.9863
0.3	0.7	21	92.7835	0.9233	0.9864
0.4	0.6	21	92.7564	0.9230	0.9861
0.6	0.4	21	92.6982	0.9225	0.9865
0.0	1.0	21	92.6555	0.9222	0.9868
0.7	0.3	21	92.3896	0.9193	0.9860
1.0	0.0	21	91.6542	0.9115	0.9827
0.9	0.1	21	91.6154	0.9111	0.9829
0.8	0.2	21	91.4427	0.9095	0.9826

Based on validation-based sensitivity analysis, α = 0.2 and β = 0.8 were selected as the final fusion coefficients. The selection prioritized validation Macro-F1 to account for class imbalance, while validation accuracy and ROC-AUC were considered as secondary performance indicators. Based on the α-β sensitivity analysis, α = 0.2 and β = 0.8 was selected as the final fusion configuration because it achieved the highest validation Macro-F1 (0.9252) and ROC-AUC (0.9870) among the evaluated weight combinations. The number of retained features in this sensitivity experiment was determined under the corresponding threshold-selection procedure and is not treated as the final feature count. The evaluated α-β configurations exhibited similar feature-retention levels under their respective threshold-selection procedures, while their validation performance varied according to the relative contribution of statistical and model-based feature importance.

The sensitivity results demonstrate that AFI-ID remains relatively stable across a broad range of α-β configurations, although performance varies according to the relative contribution of statistical and model-based feature importance. The configuration α = 0.2 and β = 0.8 achieved the highest validation Macro-F1 (0.9252) and ROC-AUC (0.9870), while the α = 0.1 and β = 0.9 configuration produced a marginally higher validation accuracy (92.9659%). Because Macro-F1 provides a more appropriate measure under class imbalance, α = 0.2 and β = 0.8 was selected as the final fusion configuration. The held-out test set was not used for selecting α or β.

#### 3.2.5 τ sensitivity analysis

To evaluate the sensitivity of feature retention to the adaptive threshold, a validation-based τ sensitivity analysis was conducted. The adaptive threshold τdetermines the minimum normalized hybrid feature score required for a feature to be retained. For a candidate threshold τ∈T, the retained feature subset is defined as given in Equation 7:


$$\begin{array}{c} F_{\tau }=\left\{f_{i}\in F\;|\;S_{hybrid}\left(f_{i}\right)\geq \tau \right\} \end{array}$$ (7)

The optimal threshold is selected using the validation Macro-F1, as defined in Equation 8:


$$\begin{array}{c} \tau^{*}=\arg\underset{\tau \in T}{\max}F1_{macro}^{val}\left(F_{\tau }\right). \end{array}$$ (8)

Here, T denotes the predefined candidate threshold set, and $F1_{macro}^{val}\left(F_{\tau }\right)$ denotes the Macro-F1 obtained on the validation subset using the feature subset *F*_τ_. When multiple candidate thresholds produce comparable validation Macro-F1, the threshold retaining fewer features is preferred. The held-out test set is not used during threshold selection.

Thus, τ^*^ is not a manually fixed threshold. It is selected from the candidate threshold set according to validation performance, and the corresponding feature subset $F_{\tau^{*}}$ is used for subsequent base-learner training. Consequently, the number of retained features is determined by the empirical distribution of the hybrid feature scores and the validation response to each candidate threshold, rather than by prescribing a fixed feature count.

The adaptive threshold is selected through a validation-based search over the candidate set T={0.10,0.15,0.20,0.25,0.30,0.35,0.40,0.45,0.50}. For each candidate threshold τ, features satisfying *S*_*hybrid*_(*f*_*i*_) ≥ τ are retained, and the resulting feature subset is evaluated using the complete AFI-ID classification pipeline. The optimal threshold is determined according to the maximum validation Macro-F1, with the number of retained features used as a secondary criterion in the case of a tie. For UNSW-NB15, τ^*^ = 0.10 achieved the highest validation Macro-F1 of 0.9270, with a validation accuracy of 93.2085% and ROC-AUC of 0.9864. This threshold retained 17 features.

At the selected threshold, the lowest-scoring retained feature was ct_src_dport_ltm with a normalized hybrid score of 0.117853, whereas the next-ranked feature, dbytes, had a score of 0.099322 and was excluded. Thus, the threshold provides an explicit data-dependent boundary for the final feature subset.

As shown in Table 5, τ = 0.10 provides the highest validation Macro-F1 (0.9270), together with a validation accuracy of 93.2085% and ROC-AUC of 0.9864. Accordingly, the final AFI-ID configuration for UNSW-NB15 uses τ = 0.10 and retains 17 features.

**Table 5:** Sensitivity analysis of the adaptive threshold τ for UNSW-NB15.

Threshold τ	Selected features	Validation accuracy (%)	Validation Macro-F1	Validation ROC-AUC
0.10	17	93.2085	0.9270	0.9864
0.15	12	92.9885	0.9247	0.9860
0.20	5	89.3405	0.8753	0.9385
0.25	3	88.7791	0.8693	0.9229
0.30	3	88.7791	0.8693	0.9229
0.35	3	88.7791	0.8693	0.9229
0.40	2	87.2164	0.8474	0.8971
0.45	2	87.2164	0.8474	0.8970
0.50	1	87.2164	0.8474	0.8347

The optimal threshold τ^*^ = 0.10 is selected according to the highest validation Macro-F1.

Features are ranked in descending order of *S*_*i*_, and a subset is selected using a data-driven adaptive threshold-based criterion instead of selecting a fixed number of features:

Specifically, only features with hybrid scores greater than or equal to τ are retained. The threshold τwas determined through validation-based sensitivity analysis across multiple candidate threshold values, each producing a corresponding feature-retention level. The final τ value was selected according to the validation-based criterion defined in Algorithm 1, prioritizing the highest validation Macro-F1 and, in the case of comparable performance, preferring the threshold that retains fewer features. To prevent information leakage, all hybrid feature scoring operations, including MI, χ^2^, XGB, and ET importance estimation, were computed exclusively using the training partition prior to model evaluation. To prevent information leakage during stacking, training meta-features were generated using 5-fold out-of-fold (OOF) predictions. Specifically, the training partition was divided into five stratified folds. For each fold, the Logistic Regression (LR), Extra Trees (ET), and XGBoost (XGB) base classifiers were trained on the remaining 4 folds and used to generate predictions for the held-out fold. The five held-out prediction sets were then concatenated to obtain OOF predictions for every training instance. For the validation and test partitions, the base classifiers were trained on the complete training partition and subsequently used to generate predictions for the corresponding partitions. Thus, no target-label information from a sample was used to generate its training meta-features. For each base classifier, the predicted class probabilities were transformed into four structured meta-features: log-probabilities for the two classes, prediction confidence, and prediction entropy. The resulting representation therefore contains 12 meta-features arranged as a sequence of three classifier-specific representations. These representations are subsequently processed by the BiLSTM with attention mechanism.

Algorithm 1 **Adaptive threshold selection in AFI-ID.**


 Input:
    Training set Dtrain
    Validation set Dval
    Hybrid feature scores Shybrid
    Candidate thresholds T
 Output:
    Optimal threshold τ^*^
    Selected feature subset F^*^
 1. Initialize the best validation Macro-F1 to –∞.
 2. For each τ in T:
    a. Construct Fτ = {fi : Shybrid(fi) ≥ τ}.
    b. Construct *F*_τ_, train the heterogeneous base
    learners (LR, ET, and XGB) using *F*_τ_, generate
    the corresponding structured meta-features, and
    train/evaluate the attention-based BiLSTM meta-
    classifier on the validation subset.
    c. Calculate validation Macro-F1 on Dval.
    d. Record the number of retained features.
 3. Select τ^*^ corresponding to the highest validation Macro-F1.
 4. If multiple thresholds yield the same validation Macro-F1, select the threshold retaining fewer features.
 5. Set F^*^ = *F*_τ_^*^, where F^*^is the dataset-specific feature subset determined by the selected threshold.
 6. Use F^*^ for subsequent base-learner and meta-classifier training.
 7. Reserve the test set exclusively for final evaluation.

### 3.3 Heterogeneous base learner

At the base (Level-0), AFI-ID employs three heterogeneous classifiers, namely, LR, ET, and XGB. These models are selected to ensure diversity across linear, bagging-based, and boosting-based learning paradigms. This heterogeneity improves ensemble generalization and reduces correlated prediction errors.

Let the filtered feature subset obtained from Section 3.2 be represented by F^*^ ∈ ℝ^*n* × *d*^. Each base learner independently learns a mapping:


$$\begin{array}{c} f_{m}:F^{*}\to Ŷ,m\in \left\{LR,ET,XGB\right\}\; \end{array}$$

where $\hat{Y\;}$ denotes the predicted class probability outputs.

The LR model is a traditional discriminative method widely used in binary classification of intrusion detection systems (Buczak and Guven, 2016). It is a probability model of a binary outcome, conditioned on the occurrence of another binary outcome through the sigmoid function, as defined in Equation 9:


$$\begin{array}{c} P\left(y=1\;|\;x\right)=\frac{1}{1+e^{-\left(w^{T}x+b\right)}} \end{array}$$ (9)

In which *w* ∈ ℝ^*d*^ is the weight vector, *b* ∈ ℝ is the bias term, and *x* ∈ ℝ^*d*^ is the input feature vector.

The binary cross-entropy loss is minimized to optimize LR parameters, as defined in Equation 10:


$$\begin{array}{c} L_{LR}=-\frac{1}{n}\overset{n}{\underset{i=1}{\sum }}\left[y_{i}\;\log{ŷ}_{i}+\left(1-y_{i}\right)\log\left(1-{ŷ}_{i}\right)\right] \end{array}$$ (10)

LR provides stable predictions and improves interpretability within the ensemble.

ET classifier is an ensemble of decision trees, based on randomized choice, which, in contrast to other ensemble classifiers, uses all of the training set (no bootstrapping) and randomly picks the split thresholds. The majority voting is used to obtain ensemble predictions, as defined in Equation 11:


$$\begin{array}{c} F\left(x\right)=\mathrm{mode}\left(f_{1}\left(x\right),f_{2}\left(x\right),\ldots ,f_{K}\left(x\right)\right) \end{array}$$ (11)

where *K* denotes the total number of trees in the ET ensemble and *fk*(*x*) represents the prediction of the *k*-th decision tree. This stochastic splitting strategy increases diversity among the trees, which reduces variance and improves robustness to noise.

XGB is a gradient-boosting model that builds regression trees one after another to reduce a differentiable loss.

The *t*−*th* iteration has the objective defined in Equation 12:


$$\begin{array}{c} L^{\left(t\right)}=\overset{n}{\underset{i=1}{\sum }}l\left(y_{i},{ŷ}_{i}^{\left(t\right)}\right)+\overset{t}{\underset{k=1}{\sum }}\Omega \left(f_{k}\right) \end{array}$$ (12)

where $l\left(y_{i},{ŷ}_{i}^{\left(t\right)}\right)$ is the training loss and Ω(*f*_*k*_) is the regularization term defined in Equation 13:


$$\begin{array}{c} \Omega \left(f\right)=\gamma T+\frac{1}{2}\lambda \overset{T}{\underset{j=1}{\sum }}w_{j}^{2} \end{array}$$ (13)

Where, *T* is the number of leaves, *w*_*j*_ is the weight of leaves, γ is used to penalize the complexity of a tree, and λ is used to control L2 regularization. XGB is able to capture non-linear interactions and performs effectively on high-dimensional and imbalanced intrusion detection data. The predictions generated by the base learners are retained in probabilistic form and sent to the structured meta-feature extraction module.

### 3.4 Meta-feature extraction and attention-based stacking

Unlike traditional stacking models that directly combine predicted labels or probabilities, AFI-ID produces structured meta-features that capture the characteristics of confidence and uncertainty of the classifier.

#### 3.4.1 Meta-feature construction

For each sample *i* and each base model *m*, class-wise probability representations are extracted and transformed into structured meta-features for the stacking framework. For multiclass classification, the predicted probability vector is represented as:


$$\begin{array}{c} p_{i}\;=\;\left[p_{i}^{1},\;p_{i}^{2},\;\ldots ,\;p_{i}^{C}\right]\; \end{array}$$

where *c* denotes the total number of classes, $p_{i}^{c}$ represents the predicted probability for class *c*.

The corresponding class-wise log-probability representation is computed as defined in Equation 14:


$$\begin{array}{c} z_{i}^{c}\;=\;log\left(p_{i}^{c}\right) \end{array}$$ (14)

where $z_{i}^{c}$ denotes the log-probability score associated with class *c* for sample *i*.

For multiclass classification, the complete vector of class-wise log-probability features is provided to the meta-classifier, enabling structured meta-feature construction across multiple intrusion categories.

The confidence score for each base learner is defined in Equation 15:


$$\begin{array}{c} c_{i}^{m}=max_{k}p_{i,\;k}^{m} \end{array}$$ (15)

The entropy-based uncertainty measure is defined in Equation 16:


$$\begin{array}{c} e_{i}^{m}=\;-\underset{k}{\sum }p_{i,\;k}^{m}\log p_{i,\;k}^{m} \end{array}$$ (16)

The meta-feature vector for sample *i* is thus:


$$\begin{array}{c} \phi_{i}=\left[z_{i}^{LR},c_{i}^{LR},e_{i}^{LR},z_{i}^{ET},c_{i}^{ET},e_{i}^{ET},z_{i}^{XGB},c_{i}^{XGB},e_{i}^{XGB}\right] \end{array}$$

The resulting meta-feature tensor is represented as: Φ ∈ ℝ^*n*×*T*×*f*^

Each sample is denoted by n, *T*= 3 the number of base models, and *f* denotes the dimensionality of the extracted meta-feature representation for each base learner.

#### 3.4.2 Attention-based BiLSTM meta-classifier

A BiLSTM network is used to learn inter-classifier relationships, as it effectively captures contextual dependencies within sequential representations, as defined in Equation 17 (Zhang et al., 2023):


$$\begin{array}{c} h_{t}=\text{BiLSTM}\left(\phi_{t};\theta \right) \end{array}$$ (17)

where *h*_*t*_ denotes the hidden representation at step *t* and θ represents the trainable parameters of the BiLSTM network. Unlike shallow aggregation strategies such as conventional MLP-based stacking, the proposed BiLSTM-based architecture models contextual dependencies among heterogeneous classifier-generated meta-features through bidirectional sequential representation learning. This enables improved attention-guided aggregation, multiclass robustness, and minority-class sensitivity under heterogeneous intrusion patterns.

The architecture further incorporates an attention mechanism that assigns attention-based importance weights to the outputs of the base learners, thereby improving attention-guided aggregation capability and minority-class sensitivity, with the attention weights defined in Equation 18 (Singh et al., 2025):


$$\begin{array}{c} \alpha_{t}=\frac{\exp\left(w_{a}^{T}\tanh\left(W_{h}h_{t}\right)\right)}{\;}\overset{T}{\underset{j=1}{\sum }}\exp\left(w_{a}^{T}\tanh\left(W_{h}h_{j}\right)\right) \end{array}$$ (18)

where *W*_*h*_ denotes the trainable attention projection matrix and *w*_*a*_ represents the trainable attention weight vector. Context vector is then calculated as defined in Equation 19:


$$\begin{array}{c} c=\overset{T}{\underset{t=1}{\sum }}\alpha_{t}h_{t} \end{array}$$ (19)

where *c* denotes the attention-weighted context vector summarizing the contributions of all base learners. Final prediction is obtained as defined in Equation 20:


$$\begin{array}{c} ŷ=softmax\left(W_{c\;}\;c\right) \end{array}$$ (20)

where *Wc* denotes the output projection matrix and *y* represents the final class probability prediction. Unlike conventional stacking models that directly operate on predicted probabilities, AFI-ID integrates hybrid feature intelligence with confidence-aware attention-based fusion within a coordinated sequential pipeline. This coordinated design improves discriminative capability and enhances sensitivity toward minority intrusion classes. The effectiveness of this integrated architecture is validated through extensive experimental evaluation in the following section.

Hyperparameter tuning was performed using grid search on the training data, with model selection based on validation performance. The dataset was split into training and validation subsets, and multiple hyperparameter combinations were systematically evaluated.

For LR, the regularization strength (C) was searched over [0.01, 0.1, 1, 10], and the solver was selected from {liblinear, lbfgs}. For ET, the number of estimators was varied in [50, 100, 200], and maximum tree depth in {None, 10, 20}. For XGB, the number of estimators was tuned over [50, 100, 200], learning rate over [0.01, 0.1, 0.2], and maximum depth over [3, 6, 9].

For the BiLSTM meta-classifier, the number of hidden units was explored in [32, 64, 128], dropout rate in [0.1, 0.2, 0.3], batch size in [16, 32, 64], and training epochs in [20, 30, 40]. The Adam optimizer was used with a learning rate of 10^{−3 }^.

The final hyperparameter values reported in Table 3 correspond to the configurations that achieved the best validation performance. This systematic tuning ensures reproducibility while maintaining computational efficiency. Although several components used in the proposed framework, such as preprocessing techniques, classical machine learning classifiers, and deep learning architectures, are well-established in the literature, The primary contribution of AFI-ID lies in the coordinated sequential integration of hybrid feature intelligence and attention-based ensemble aggregation within a coordinated intrusion detection framework.

Specifically, the novelty of this work is twofold: (i) the development of a hybrid adaptive feature intelligence mechanism that combines statistical relevance and model-based importance through a weighted hybrid fusion strategy, and (ii) the design of an attention-based deep stacking architecture that enables attention-guided aggregation of heterogeneous base learners using structured meta-features.

Unlike conventional intrusion detection systems where feature selection and ensemble aggregation are treated as independent stages, AFI-ID establishes a tightly coupled interaction between feature representation refinement and classifier decision-making. This coordinated design contributes to improved discriminative capability, enhanced robustness under class imbalance, and stronger generalization across heterogeneous intrusion scenarios.

Therefore, the primary contribution of AFI-ID lies in the coordinated integration of hybrid feature intelligence and attention-based ensemble aggregation within a unified intrusion detection framework.

## 4 Experimental setup

The empirical evaluation of AFI-ID is designed to assess its discriminative capability, robustness to class imbalance, and stability across repeated training runs. Three aspects are specifically addressed in the experimental design: (i) feature compactness and predictive power when the proposed AFI mechanism is applied; (ii) the efficacy of structured attention-based stacking in enhancing ensemble decision-making; and (iii) consistency in performance when performing binary and multiclass intrusion detection tasks.

All experiments were implemented in Python, using Scikit-learn (Inria, Paris, France) for traditional machine learning models and PyTorch (Meta Platforms, Inc., Menlo Park, CA, USA) for the deep meta-classifier. The Level-0 learners include LR, ET (Geurts et al., 2006), and the XGB (Chen and Guestrin, 2016). These models have been selected deliberately to depict linear, bagging based and boosting based learning paradigms, thus encouraging the variety of classifiers in the stacking architecture. This heterogeneity improves ensemble generalization performance in IDS (Bibers et al., 2024).

To address class imbalance, as described in Section 3.1, SMOTE was applied only to the training partition. The datasets used in the evaluation were not manipulated in the training process to maintain the statistical validity of the evaluation and avoid the information leakage. All experiments were conducted using identical preprocessing pipelines and evaluation protocols across baseline and proposed models to ensure fair comparison.

### 4.1 Experimental datasets

Two benchmark intrusion detection datasets were used to evaluate the proposed framework: UNSW-NB15 (Moustafa and Slay, 2015) and CICIDS2017 (Sharafaldin et al., 2018). These datasets provide diverse attack categories and heterogeneous traffic patterns, enabling comprehensive evaluation under both binary and multiclass classification settings.

The UNSW-NB15 dataset was introduced to address the limitations of earlier datasets such as KDDCup99 and NSL-KDD, which suffer from redundant records and outdated traffic characteristics. It incorporates modern attack scenarios along with realistic background traffic, making it suitable for evaluating machine learning-based intrusion detection systems. The dataset comprises 42 features representing protocol behavior, statistical flow properties, content-level attributes, and connection-based characteristics. It includes nine attack categories—Analysis, Backdoor, DoS, Exploits, Fuzzers, Generic, Reconnaissance, Shellcode, and Worms—along with normal traffic. Table 6 presents the class-wise distribution of the UNSW-NB15 dataset used in this study.

**Table 6:** Class-wise distribution of UNSW-NB15.

Class	Training	Evaluation	Total
Normal	56,000	37,000	93,000
Generic	40,000	18,871	58,871
Exploits	33,393	11,132	44,525
Fuzzers	18,184	6,062	24,246
DoS	12,264	4,089	16,353
Reconnaissance	10,491	3,496	13,987
Analysis	2,000	677	2,677
Backdoor	1,746	583	2,329
Shellcode	1,133	378	1,511
Worms	130	44	174
Total	175,341	82,332	257,673

The dataset contains 175,341 training samples and 82,332 testing samples, totaling 257,673 records. The predefined training–testing split is preserved to maintain consistency with established evaluation protocols and to reduce potential information leakage effects.

To further evaluate cross-dataset generalization, experiments were also conducted on the CICIDS2017 dataset (Sharafaldin et al., 2018), which reflects contemporary enterprise network behavior and includes modern attack vectors alongside realistic background traffic. CICIDS2017 provides flow-based network features capturing temporal statistics, packet length distributions, header information, and protocol-level characteristics. Following the data-cleaning and feature-removal procedures described below, 77 input features were retained for the present experiments.

The CICIDS2017 dataset contains flow-based network traffic representing benign and attack activities. In the present study, the eight available CICIDS2017 CSV files were loaded and subjected to the described data-cleaning procedures. Infinite values were replaced with missing values, rows containing missing values were removed, labels were converted into a binary representation (BENIGN = 0 and attack = 1), and Flow ID, Timestamp, Source, Destination, Protocol, and Port-related fields were removed. After these procedures, the resulting dataset comprised 2,827,876 flow records with 77 input features, including 2,271,320 benign samples (80.3189%) and 556,556 attack samples (19.6811%). For the AFI-ID experiments, a stratified experimental subset of the cleaned CICIDS2017 data was used to reduce computational requirements while preserving the dominant benign-to-attack distribution. The experimental subset contained 282,787 flow records and was subsequently divided into training, validation, and testing partitions using a 60:20:20 ratio. The resulting partitions contained 169,672 training samples, 56,557 validation samples, and 56,558 testing samples. The resulting experimental partition is summarized in Table 7. The subsequent training, validation, and testing procedures were performed on this experimental subset. Because the sampling procedure cannot guarantee sufficient representation of extremely rare attack categories, categories with very limited original support, such as Heartbleed and Web Attack–SQL Injection, may remain underrepresented or absent in individual evaluation partitions. Therefore, the results for such rare categories are interpreted cautiously and are not considered representative of their full-dataset behavior.

**Table 7:** CICIDS2017 experimental partition.

Partition	Total samples	Benign	Attack	Proportion
Training	169,672	~136,274	~33,398	60%
Validation	56,557	~45,425	~11,132	20%
Testing	56,558	~45,426	~11,132	20%
Total	282,787	~227,125	~55,662	100%

Despite its widespread adoption, CICIDS2017 has known limitations that may affect model evaluation. First, the labeling process is based on predefined attack scenarios and flow-level aggregation, which may introduce label noise, particularly in cases of overlapping or ambiguous traffic patterns. Second, temporal and session-level dependencies among flows create a potential risk of information leakage when flows originating from the same underlying attack scenario or communication session are distributed across different data partitions. Such dependencies can lead to optimistic estimates of generalization performance.

The evaluation across UNSW-NB15 and CICIDS2017 demonstrates the behavior of AFI-ID under two heterogeneous benchmark distributions; however, it does not establish robustness to temporal distribution shift or concept drift in operational network environments.

### 4.2 Demonstration workflow for reproducibility

To improve transparency and facilitate practical understanding of the proposed AFI-ID framework, a simplified demonstration workflow is provided using a representative subset of the benchmark datasets. For demonstration purposes, a small stratified sample can be extracted from the UNSW-NB15 or CICIDS2017 datasets to preserve class distribution while reducing computational overhead.

The demonstration follows the complete AFI-ID pipeline: (i) data preprocessing, including missing value handling, label encoding of categorical features, and Min–Max normalization; (ii) hybrid adaptive feature intelligence, where statistical relevance (MI and χ^2^) and model-based importance (XGB and ET) are combined to select a reduced feature subset; (iii) training of base classifiers such as Logistic Regression, Extra Trees, and XGB; and (iv) attention-based meta-classification using a BiLSTM model to dynamically aggregate base learner outputs.

This step-by-step workflow improves transparency and enables reproducibility of the proposed AFI-ID framework on smaller representative subsets. Since the framework relies on standard preprocessing techniques and widely available machine learning models, it can be easily implemented in common environments such as Python with Scikit-learn and PyTorch. The use of publicly available datasets further ensures that the proposed method can be reproduced and validated by other researchers. The datasets used in this study are publicly available, and additional data availability details are provided before the References section to further support reproducibility.

### 4.3 Implementation environment and training setup

The proposed AFI-ID framework was developed and evaluated on a local workstation equipped with an Intel i7 processor (3.4 GHz), 16GB system memory and an NVIDIA GTX 1660 graphics card running the Ubuntu 22.04 operating system. Python 3.10 (Python Software Foundation, Wilmington, DE, USA), Scikit-learn 1.3, PyTorch 2.0, and XGBoost 1.7 [XGBoost open-source project (DMLC), USA] were included in the software environment. Unless otherwise stated, all preprocessing, data partitioning, feature selection, oversampling, and model training procedures were initialized using a fixed random seed of 42 to ensure experimental reproducibility. Additional robustness analysis was subsequently conducted using multiple random seeds, as reported in Section 5.1.3.

Hyperparameter tuning was conducted using controlled grid-search exploration. In the case of Logistic Regression, the regularization coefficient was changed over a range of different magnitudes to control the smoothness of the decision boundaries. The tree-based learners were tuned by varying the maximum tree depth and ensemble size to balance bias and variance. The XGB parameters were chosen through the simultaneous optimization of the tree depth, learning rate, and boosting rounds to achieve a stable convergence.

To prevent overfitting, the attention fusion module was set to use a 64-dimensional hidden representation and dropout regularization value of 0.2. Adam was used with a learning rate of 10^−3^ and mini-batches of 32. Although explicit early stopping was not employed, the convergence analysis demonstrated stable validation performance without significant divergence from the training curve, indicating limited epoch-wise overfitting during BiLSTM training.

Under this configuration, the base learners converged efficiently, requiring approximately 1 min to reach convergence. The deep stacking architecture introduced additional computational overhead; as it involved sequential meta-feature construction and attention-based optimization, while inference remained computationally feasible for offline intrusion analysis.

### 4.4 Evaluation metrics and validation protocol

Confusion-matrix-based evaluation metrics were used to assess both overall predictive performance and class-wise discrimination capability. Since intrusion detection datasets naturally exhibit severe class imbalance, the evaluation is not only based on aggregate correctness but also on the balance between false alarms and misdetections. Here, TP, FP, TN, and FN denote true positive, false positive, true negative, and false negative instances, respectively. For binary intrusion detection, the total accuracy is calculated as:


$$\begin{array}{c} \text{Accuracy}=\frac{\text{TP}+\text{TN}}{\text{TP}+\text{TN}+\text{FP}+\text{FN}} \end{array}$$

However, accuracy alone can be misleading when evaluating minority classes in highly imbalanced datasets. Thus, precision and recall are considered together. Precision and Recall are used to measure the reliability of positive predictions and the ability to detect genuine attack instances, respectively.


$$\begin{array}{c} \text{Precision}=\frac{\text{TP}}{\text{TP}+\text{FP}} \end{array}$$

$$\begin{array}{c} \text{Recall}=\frac{\text{TP}}{\text{TP}+\text{FN}} \end{array}$$

To provide a balanced evaluation of these two measures, the harmonic mean of the F1-score is determined:


$$\begin{array}{c} \text{F}1-\text{score}=\frac{2\cdot \text{Precision}\cdot \text{Recall}}{\text{Precision}+\text{Recall}} \end{array}$$

For multiclass evaluation, the metrics are calculated on a per-class basis using a one-vs.-rest formulation. Where necessary, the scores are reported as macro-averaged and weighted-average scores to represent performance of dominant and minority-type attacks.

Threshold-independent discrimination capability is analyzed using ROC curves. In binary classification, ROC curves are used to show the trade-off between the true positive rate and the false positive rate with respect to the decision threshold. The multiclass approach uses ROC analysis; in this case a one-vs.-rest strategy is used and performance curves are shown both class-wise and micro-averaged. The Area Under the Curve (AUC) provides a scalar measure of class separability.

In order to test resilience with respect to sampling variability, experiments were repeated using multiple random seeds. Stability was evaluated using the mean accuracy and standard deviation across repeated runs. This analysis provides insight into the robustness of the proposed AFI-ID framework.

To account for class imbalance, evaluation was performed using complementary threshold-dependent and threshold-independent metrics. Accuracy measures the overall proportion of correctly classified instances, whereas Macro-F1 is calculated as the unweighted mean of the class-wise F1-scores, thereby assigning equal importance to minority and majority classes. Balanced Accuracy is calculated as the mean recall across classes, while Matthews Correlation Coefficient (MCC) provides a correlation-based measure of agreement between predicted and true labels. For binary classification, PR-AUC is calculated from the precision–recall curve using the intrusion-class probability. For multiclass classification, macro-PR-AUC is computed using a one-vs.-rest formulation across classes. ROC-AUC is reported using the corresponding one-vs.-rest formulation for multiclass evaluation, with micro-averaged ROC-AUC retained to characterize aggregate discrimination across all instances. Per-class Precision, Recall, and F1-score are also reported to explicitly assess performance for individual attack categories.

In the case of UNSW-NB15, the training and testing partitions were already defined to maintain consistency with established benchmarking protocols. In CICIDS2017, stratified splitting was used to preserve proportional class representation. Synthetic oversampling using SMOTE was applied exclusively to the training subset to prevent information leakage and preserve the integrity of model evaluation. Unlike conventional pipelines where feature selection and ensemble aggregation are treated as independent processes, AFI-ID integrates these components within a coordinated sequential learning pipeline. The feature-selection stage is optimized using validation-based selection, and the resulting feature representation is subsequently used by the ensemble-learning stage.

## 5 Findings and performance review

### 5.1 Performance of hybrid feature selection

The proposed Hybrid AFI module is evaluated through feature-count sensitivity, feature-importance analysis, and threshold sensitivity analysis. This demonstrates that the hybrid scoring mechanism produces a compact and highly discriminative feature subset prior to ensemble aggregation. The relationship between the number of selected features and validation accuracy is illustrated in Figure 6.

**Figure 6:**
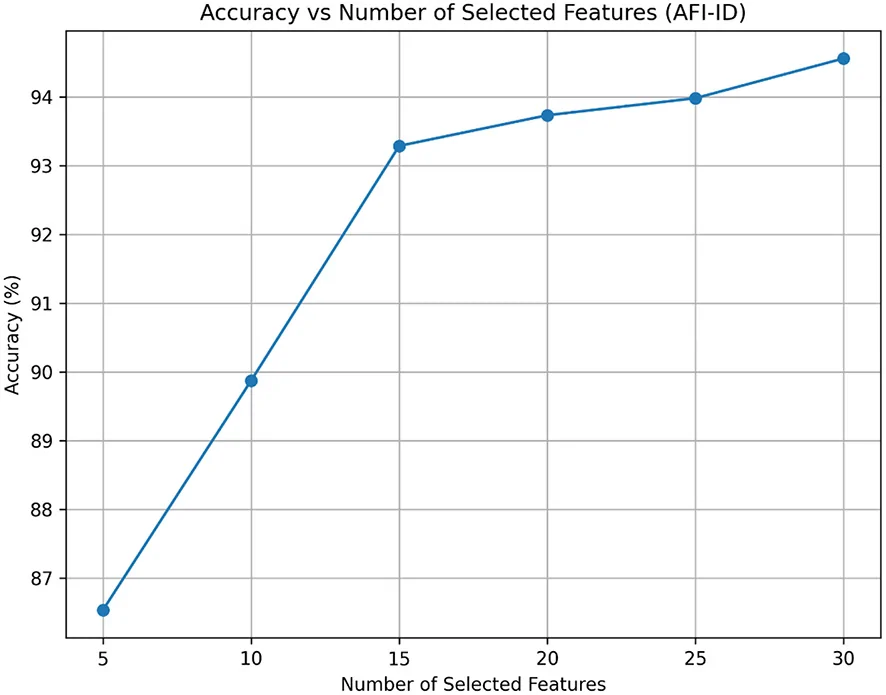
Accuracy vs. number of selected features using AFI-ID.

The results show that classification accuracy improves substantially as the number of selected features increases from 5 to 15. Specifically, validation accuracy increases from 86.55% with 5 features to 89.90% with 10 features and 93.30% with 15 features. Beyond 15 features, accuracy continues to improve, reaching 93.75%, 94.00%, and 94.55% with 20, 25, and 30 features, respectively, while the incremental improvement remains relatively small. This indicates that a substantial proportion of the discriminative information is captured within a relatively compact feature subset. However, the final feature subset is not selected solely on the basis of this fixed feature-count analysis. Instead, the subsequent validation-based adaptive-threshold procedure determines the retained features according to the hybrid feature score and validation Macro-F1.

The subsequent threshold sensitivity analysis identifies τ^*^ = 0.10 as the selected threshold for UNSW-NB15, resulting in 17 retained features. The relationship between the number of selected features and validation accuracy is illustrated in Figure 6, and the corresponding validation accuracies for different feature counts are reported in Table 8.

**Table 8:** Validation accuracy for different numbers of selected features on UNSW-NB15.

Number of selected features	Validation accuracy (%)
5	86.55
10	89.90
15	93.30
20	93.75
25	94.00
30	94.55

The hybrid feature score is defined as:


$$\begin{array}{c} S_{i}=\;\alpha S_{i}^{\left\{filter\right\}}+\;\beta S_{i}^{\left\{wrapper\right\}},\;\alpha +\;\beta =\;1 \end{array}$$

where the filtering component combines Mutual Information (MI) and Chi-square statistics, while the model-based component estimates feature importance using XGB and ET models.

To make the heterogeneous feature-selection criteria comparable, MI, χ^2^, XGB, and ET importance values were independently normalized to the [0, 1] range before calculating the corresponding filter and model-based components. The final hybrid score was then obtained by weighting the normalized filter and model-based components using α = 0.2 and β = 0.8, as determined through the validation-based α-β sensitivity analysis. Therefore, the importance values reported in Tables 9, 10 and visualized in Figures 5, 7 are normalized hybrid feature-importance scores rather than raw MI, χ^2^, XGB, or ET importance values. The normalization is performed independently within each dataset so that the resulting hybrid scores are comparable for feature ranking and threshold-based retention within that dataset. The scores are normalized relative to the maximum hybrid score within each dataset for interpretability.

**Table 9:** Top selected features—UNSW-NB15.

Rank	Feature name	Normalized hybrid importance score
1	sttl	1.0000
2	dttl	0.4995
3	ct_state_ttl	0.3638
4	rate	0.2334
5	swin	0.2093
6	ct_dst_sport_ltm	0.1898
7	smean	0.1829
8	ct_dst_src_ltm	0.1796
9	dmean	0.1697
10	ct_srv_dst	0.1636
11	sbytes	0.1622
12	dload	0.1582
13	state	0.1438
14	dwin	0.1390
15	sload	0.1255
16	ct_srv_src	0.1244
17	ct_src_dport_ltm	0.1178

**Table 10:** Top selected features—CICIDS2017.

Rank	Feature name	Normalized importance score
1	Avg Bwd segment size	0.8625
2	Bwd packet length Std	0.8260
3	Bwd packet length mean	0.6811
4	Average packet size	0.6398
5	Max packet length	0.6247
6	Packet length Std	0.6038
7	Bwd packet length max	0.5686
8	Packet length variance	0.5122
9	Bwd packet length min	0.4462
10	Packet length mean	0.4221
11	Fwd IAT max	0.4204
12	Idle max	0.4095
13	PSH flag count	0.4084
14	Flow IAT max	0.4078
15	Fwd IAT Std	0.3775
16	Bwd header length	0.3604
17	Idle min	0.3246
18	Idle mean	0.3219
19	Init_Win_bytes_forward	0.3062
20	Subflow fwd bytes	0.2726
21	Total length of Fwd packets	0.2675

**Figure 7:**
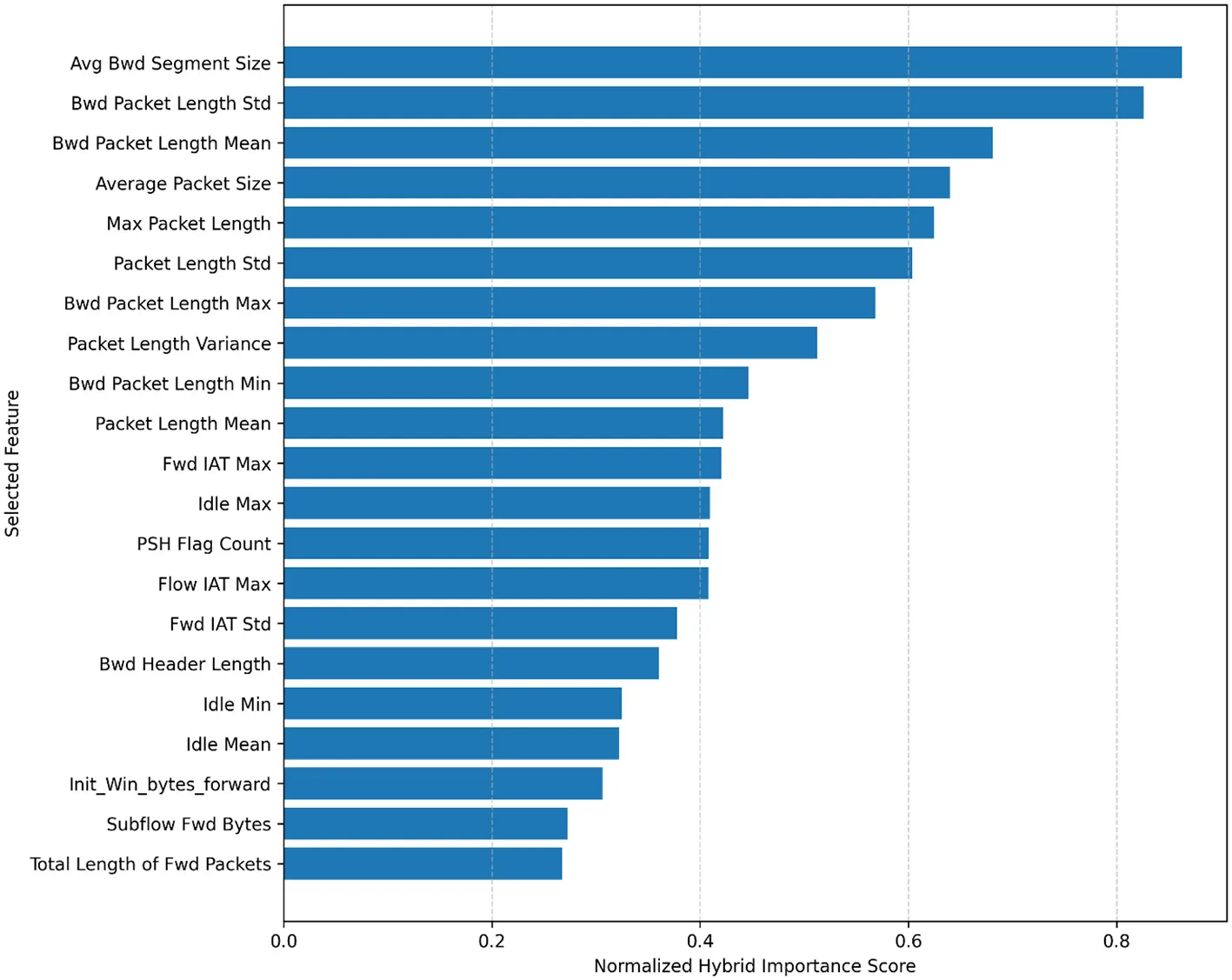
Normalized hybrid feature importance distribution—CICIDS2017.

The dashed horizontal line denotes the adaptively selected threshold τ^*^ = 0.10; features satisfying *S*_*hybrid*_(*f*_*i*_) ≥ 0.10 constitute the final 17-feature subset. Figure 5 shows the normalized hybrid feature-importance distribution for UNSW-NB15. The scores exhibit a pronounced long-tail pattern, with sttl obtaining the highest normalized hybrid importance score (1.000000), followed by dttl (0.499565) and ct_state_ttl (0.363895). The remaining selected features have progressively smaller scores, indicating that the discriminative contribution is concentrated within a compact subset of the feature space. Table 9 reports the same feature ranking and normalized hybrid scores used to generate Figure 5.

As shown in Table 9, 17 features satisfy the selection criterion $S_{hybrid}\left(f_{i}\right)\geq \tau^{*}=0.10$. The lowest-scoring retained feature is ct_src_dport_ltm, with a normalized hybrid importance score of 0.117853. The next-ranked feature, dbytes, has a score of 0.099322 and is therefore excluded. This boundary confirms the data-dependent nature of the adaptive threshold and explains the resulting 17-feature subset.

The highest-ranked UNSW-NB15 features include sttl, dttl, ct_state_ttl, rate, swin, and ct_dst_sport_ltm. These features capture aspects of TTL and connection-state behavior, traffic rate, transport-level window characteristics, and destination-port activity, respectively. The ranking therefore indicates that the hybrid scoring mechanism prioritizes features describing flow intensity, transport behavior, and connection-state dynamics.

As shown in Figure 7, the importance curve of CICIDS2017 has a similar long-tail distribution. Although the cleaned CICIDS2017 dataset contains 77 input features, only a relatively small subset exhibits high hybrid importance scores, supporting the need for dimensionality reduction. Table 10 (CICIDS2017) represents the top-ranked features identified by AFI-ID.

These properties are mainly packet timing statistics (IAT measurements), packet length distributions, and asymmetry in traffic in both directions. These attributes are useful in the characterization of bursts, scanning behavior, and abnormal flow behavior that are found in DDoS as well as brute-force attacks.

In both data sets the hybrid scoring mechanism has the benefit of concentrating the discriminative relevance in a small set and at the same time suppressing redundant attributes. Since the compactness and performance stability of the hybrid adaptive feature-intelligence module have been evaluated above, the following sections examine its effect on binary and multiclass intrusion-detection performance.

### 5.2 Feature subset analysis for CICIDS2017

For CICIDS2017, the feature-subset analysis further evaluates the effect of dimensionality reduction using different numbers of selected features.

In order to select the best feature subset size, experiments with several feature subsets of increasing size based on hybrid importance ranking were done on the CICIDS2017 dataset. In particular, the performance was assessed on the basis of 21 and 27 features selected. The comparative results show that AFI-ID achieves an overall accuracy of 93.54% under both feature configurations, with insignificant difference in the MCC and Kappa statistics. The close similarity in performance in these thresholds indicates that the most discriminating attributes are clustered around the first set of rankings. Adding additional features beyond the selected subset does not yield meaningful performance improvement while increasing computational cost and model complexity. This behavior supports the effectiveness of the hybrid scoring method in prioritizing informative attributes and achieving significant dimensionality reduction without compromising predictive performance. Since the original dataset consists of 80 features, the retention of only 21 features amounts to an approximate dimensionality reduction of 73.75%, and thus it can be concluded that the proposed AFI module is efficient.

### 5.3 Component-wise evaluation

To further assess the contribution of the individual components of the proposed adaptive feature-intelligence mechanism, a component-wise evaluation was conducted on UNSW-NB15. Seven feature-selection configurations were examined: MI, χ^2^, MI + χ^2^, XGB, Extra Trees, XGB + Extra Trees, and the complete MI + χ^2^ + XGB + Extra Trees configuration. For each configuration, the corresponding normalized feature scores were evaluated across the same candidate threshold set used in the adaptive threshold analysis. The threshold for each configuration was selected using validation Macro-F1, with validation accuracy and ROC-AUC reported as secondary indicators. The held-out test set was not used for feature-selection or threshold selection. The resulting validation performance and selected feature subsets for the evaluated feature-selection strategies are presented in Table 11.

**Table 11:** Component-wise evaluation of feature-selection strategies on UNSW-NB15.

Feature-selection strategy	Optimal τ	Selected features	Validation Accuracy (%)	Validation Macro-F1	Validation ROC-AUC
Extra trees	0.10	18	93.2059	0.9273	0.9871
MI + χ^2^ + XGB + extra trees	0.10	17	93.1826	0.9269	0.9866
MI + χ^2^	0.25	20	93.1903	0.9268	0.9866
MI	0.20	31	93.0610	0.9256	0.9860
χ^2^	0.10	18	93.0041	0.9243	0.9809
XGB + extra trees	0.10	10	92.5384	0.9202	0.9846
XGB	0.10	4	89.0093	0.8715	0.9364

The component-wise results show that the individual and combined feature-selection strategies provide different trade-offs between predictive performance and feature compactness. Extra Trees alone achieved the highest validation accuracy, Macro-F1, and ROC-AUC among the evaluated configurations, while retaining 18 features. The complete AFI-ID feature-selection strategy achieved comparable performance with 17 features, obtaining a validation accuracy of 93.1826%, Macro-F1 of 0.9269, and ROC-AUC of 0.9866. In contrast, XGB alone produced substantially lower validation performance despite retaining only four features. The results indicate that combining statistical relevance with model-based importance provides a competitive balance between predictive performance and feature-space compactness. Although Extra Trees alone achieved slightly higher validation metrics, the complete AFI-ID configuration achieved comparable performance while retaining one fewer feature and integrating complementary statistical and model-based evidence.

### 5.4 Stability across random seeds

Experiments were conducted five times with different random seeds (7, 21, 42, 84, and 123) to evaluate robustness against sampling variability. The statistical results of seed-wise performance of all classifiers are given in Table 12 and the trend in comparison is represented in Figure 8.

**Table 12:** Performance stability across random seeds on UNSW-NB15.

Seed	LR_mean	LR_std	ET_mean	ET_std	XGB_mean	XGB_std	AFI-ID_mean	AFI-ID_std
7	0.8649	0.0020	0.9303	0.0003	0.9330	0.0012	0.9358	0.0015
21	0.8649	0.0008	0.9306	0.0011	0.9329	0.0014	0.9365	0.0008
42	0.8652	0.0017	0.9311	0.0007	0.9331	0.0008	0.9362	0.0002
84	0.8655	0.0015	0.9301	0.0007	0.9330	0.0012	0.9364	0.0007
123	0.8653	0.0013	0.9308	0.0008	0.9332	0.0013	0.9360	0.0008

**Figure 8:**
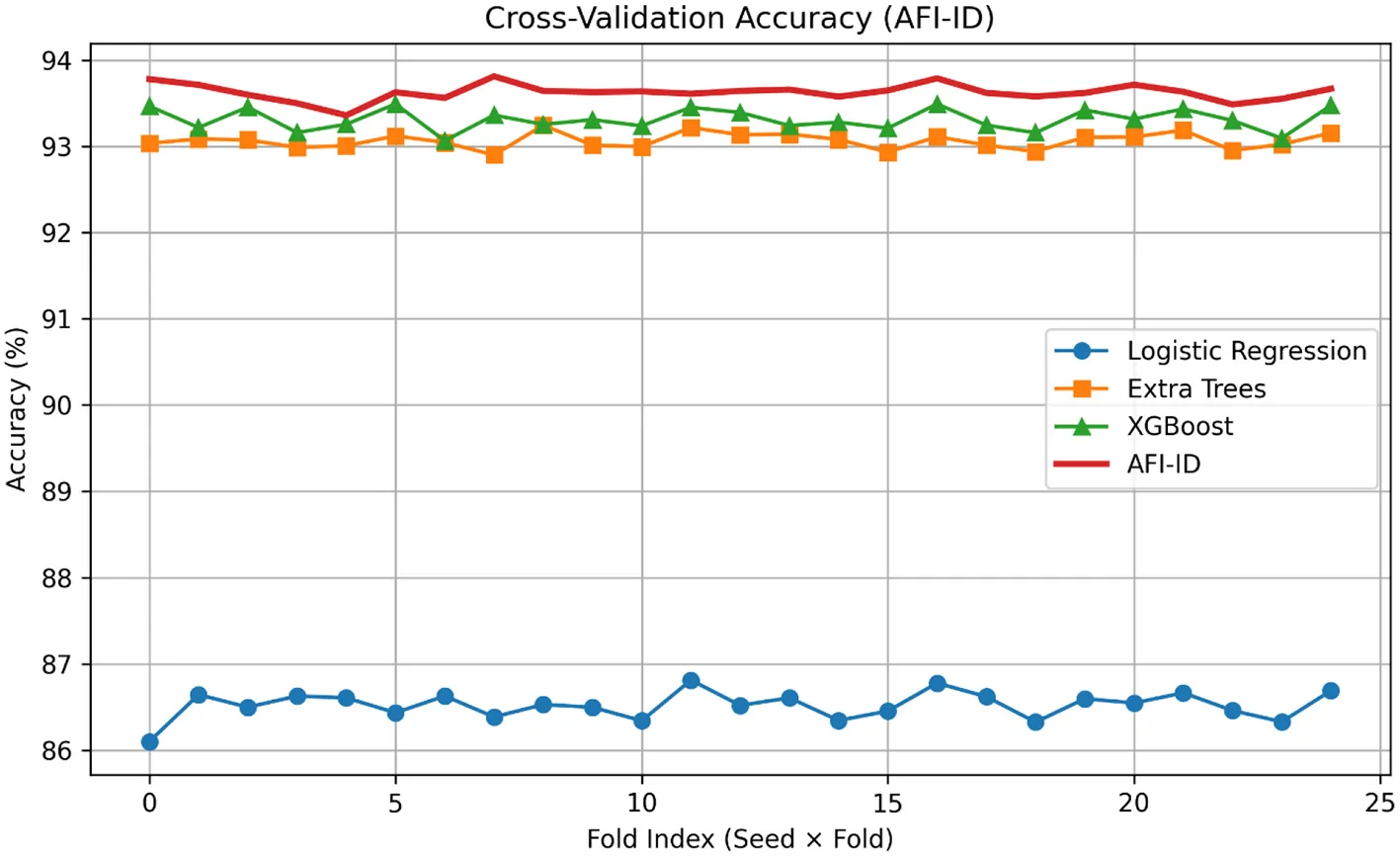
Seed-wise accuracy comparison across classifiers on UNSW-NB15.

As shown in Table 12 and Figure 8, AFI-ID consistently achieves the highest accuracy across all random seeds with minimal performance variation, demonstrating strong robustness and stability across different training splits. In contrast, the baseline models exhibit comparatively higher variability, with Logistic Regression being more sensitive to random partitioning. Across the five experimental runs, AFI-ID achieved a mean accuracy of 93.62% with a 95% confidence interval of approximately [93.57%, 93.67%], indicating stable performance and low variability.

The very low variance demonstrates that the proposed hybrid adaptive feature intelligence mechanism consistently identifies stable feature subsets across different training splits. This indicates that the selected features are not influenced by favorable sampling conditions. The repeated binary experiments provide evidence of performance stability across the evaluated random seeds.

To further validate the statistical significance of the observed performance improvements, a paired *t*-test was conducted between the proposed AFI-ID model and baseline classifiers (LR, ET, XGB) using accuracy scores obtained across multiple runs with different random seeds (Table 12). The results, presented in Table 13, show that all *p*-values are significantly below 0.05, indicating statistically significant differences. In particular, the extremely low *p*-values (on the order of 10^−5^ to 10^−10^) demonstrate that the performance improvements achieved by AFI-ID are highly unlikely to be due to random variation.

**Table 13:** Statistical significance test results (paired *t*-test on UNSW-NB15).

Comparison	*p*-value
AFI-ID vs. LR	1.08 × 10^−10^
AFI-ID vs. ET	1.38 × 10^−5^
AFI-ID vs. XGB	3.69 × 10^−5^

Prior to conducting the paired *t*-test, the distribution of performance differences across repeated runs was examined and no substantial deviation from normality was observed. Given the repeated paired evaluation design and the low variance across runs, the paired *t*-test was considered appropriate for assessing statistical significance. Statistical significance testing was performed on the repeated binary classification experiments to assess robustness across random seeds. Although consistent performance improvements were also observed in the multiclass CICIDS2017 experiments, formal significance testing for the multiclass setting was not conducted and represents an important direction for future investigation. This stability is particularly important in real-world intrusion detection scenarios, where traffic distributions may evolve over time. A model with low performance variance across different initializations is therefore more reliable for deployment in dynamic network environments. Although predefined benchmark splits were preserved to maintain consistency with established evaluation protocols, repeated experiments across multiple random seeds were additionally conducted to improve robustness assessment and reduce sensitivity to partition variability.

### 5.5 Feature analysis and interpretability

The hybrid adaptive feature intelligence mechanism selects a compact subset of discriminative features tailored to each dataset. For the UNSW-NB15 dataset, selected features predominantly include flow duration, packet size statistics, and protocol-related attributes, which are strongly associated with traffic behavior and attack patterns. In the CICIDS2017 dataset, features related to connection frequency, flow inter-arrival time, and byte rate are frequently selected, reflecting their importance in distinguishing between normal and malicious activities.

The selected features align with domain knowledge in network intrusion detection, where temporal flow characteristics and traffic intensity indicators play a critical role in identifying anomalous behavior. The hybrid selection approach effectively balances statistical relevance and model-based importance, ensuring that both global dependencies and predictive contributions are considered.

This analysis demonstrates that AFI-ID not only improves detection performance but also selects features that are meaningful and interpretable within the context of network security.

### 5.6 Binary intrusion detection

Binary intrusion detection evaluates the ability of the proposed AFI-ID framework to distinguish between benign and malicious network traffic. The models are evaluated using Accuracy, Macro-F1, Balanced Accuracy, MCC, ROC-AUC, and PR-AUC, together with per-class Precision, Recall, and F1-score and confusion-matrix analysis. These complementary metrics provide a more informative assessment than accuracy alone under class-imbalanced intrusion detection conditions.

Table 14 is the summary of binary classification on UNSW-NB15. AFI-ID achieves an overall accuracy of 93.31%, outperforming LR (86.66%), ET (91.97%), and XGB (92.43%).

**Table 14:** Binary classification performance on UNSW-NB15.

Classifier	Class	Precision	Recall	F1 score	Accuracy (%)
Logistic regression	Normal	0.8096	0.8243	0.8169	86.66
	Intrusion	0.8997	0.8905	0.8951	
Extra trees	Normal	0.8884	0.8891	0.8888	91.97
	Intrusion	0.9373	0.9369	0.9371	
XGB	Normal	0.8785	0.9171	0.8974	92.43
	Intrusion	0.9520	0.9283	0.9400	
AFI-ID	Normal	0.8953	0.9225	0.9087	93.31
	Intrusion	0.9554	0.9391	0.9472	

On the class level, the AFI-ID has a precision of 0.89538 and a recall of 0.92253 in the Normal class (F1 = 0.90875). In the case of the Intrusion class, the precision is 0.95548 and recall is 0.93912 (F1 = 0.94723). AFI-ID outperforms XGB, while maintaining comparable precision and achieving higher intrusion recall (0.93912 vs. 0.92837), which decreases the number of attacks not detected. Compared to ET, there are positive changes in both the Normal-class and Intrusion-class F1-score.

These results are further proved by the ROC comparison in Figure 9. AFI-ID has the best AUC of 0.9849, and it is better than XGB (0.9809), ET (0.9752) and LR (0.9425). This implies the improvement of threshold-independent separability between benign and malicious traffic.

**Figure 9:**
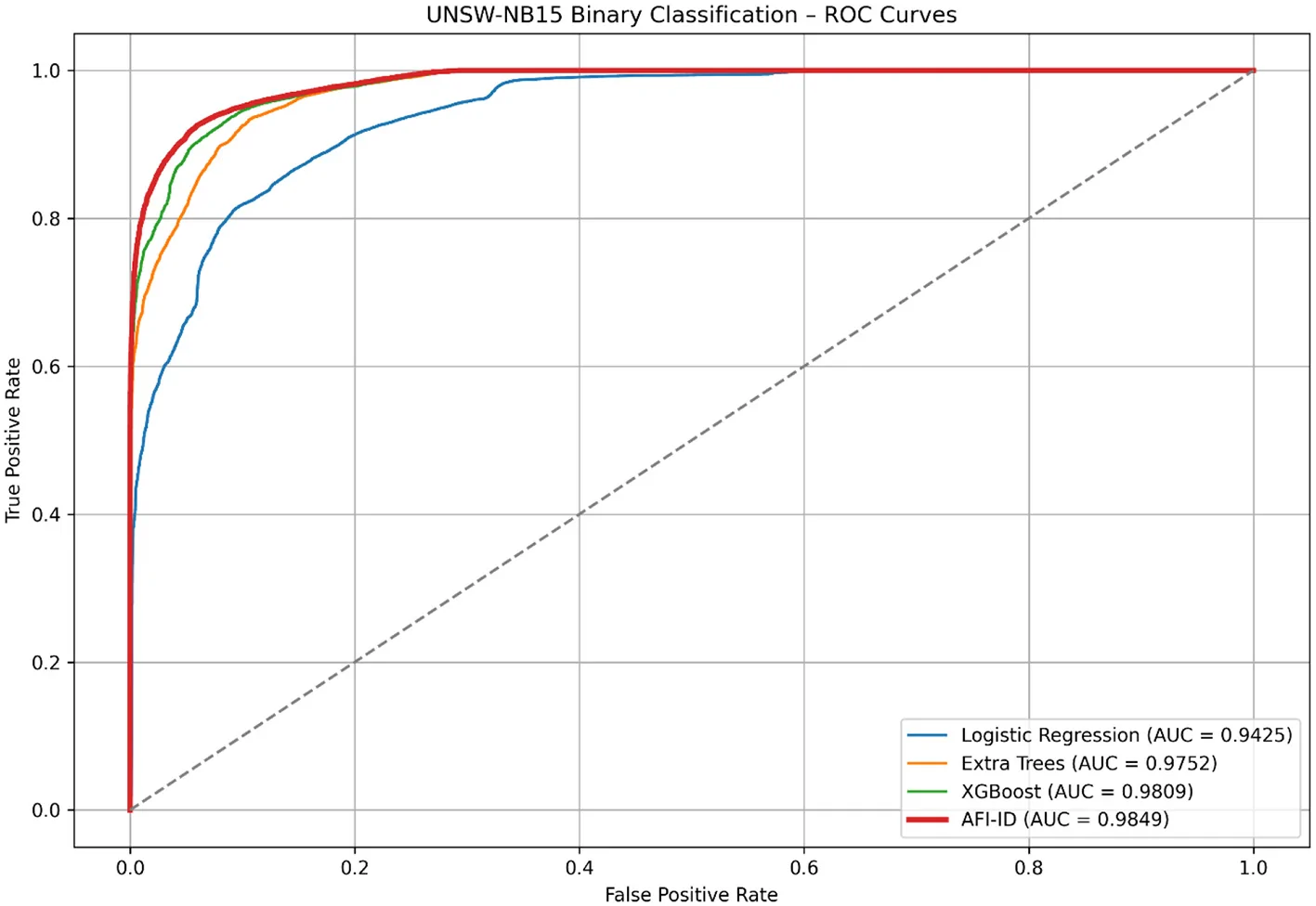
ROC curve-UNSW-NB15 (binary classification).

Further details can be seen in the confusion matrices demonstrated in Figure 10. AFI-ID minimizes the false positives (Normal misclassified as Intrusion) and false negatives (Intrusion misclassified as Normal) as compared to the baseline models. Specifically, AFI-ID produces fewer intrusion false negatives, indicating improved intrusion detection sensitivity.

**Figure 10:**
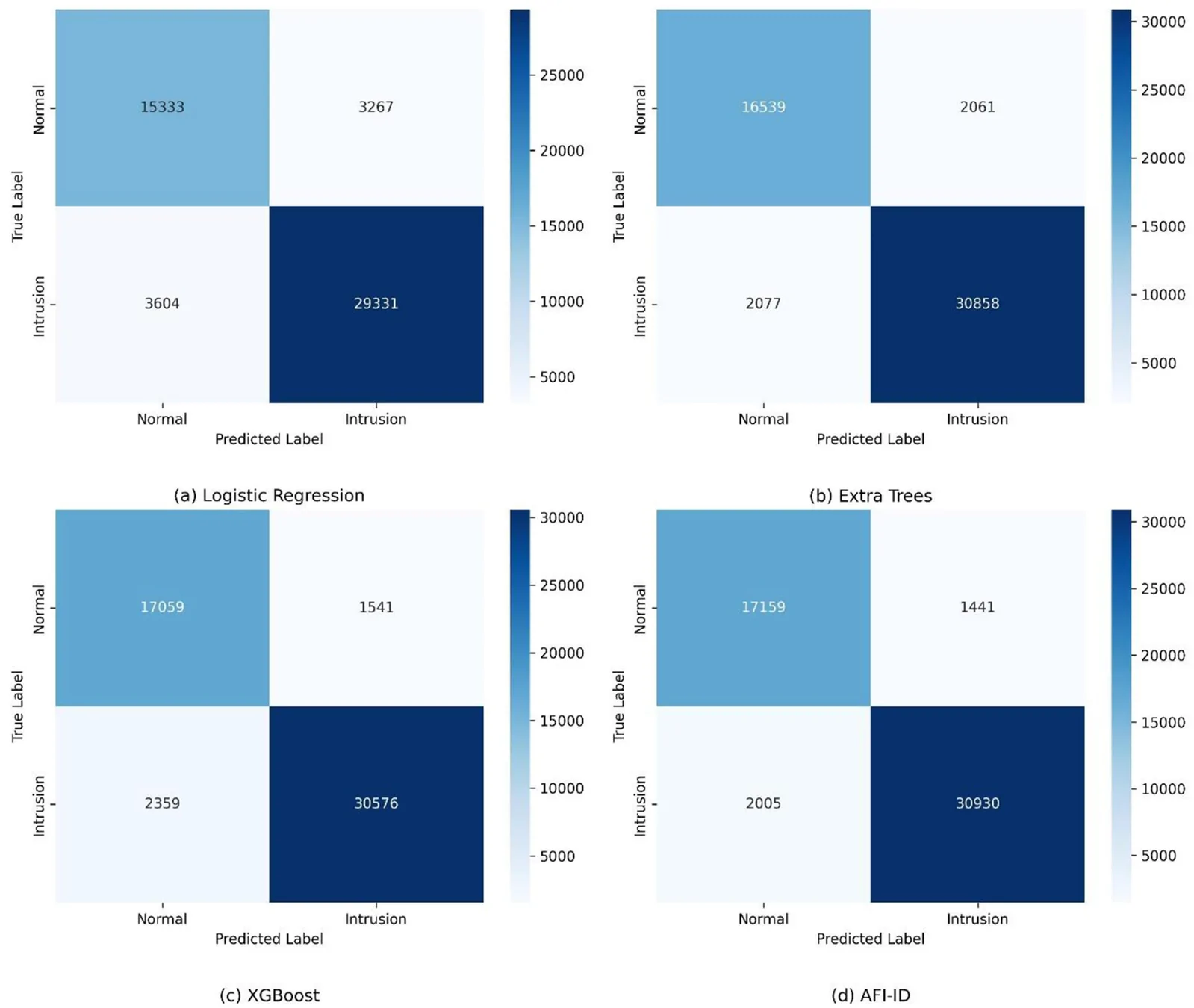
Performance comparison of the base classifiers and AFI-ID: **(a)** Logistic Regression, **(b)** Extra Trees, **(c)** XGBoost, and **(d)** AFI-ID.

Overall, the results on UNSW-NB15 indicate that AFI-ID provides improved aggregate binary detection performance relative to the evaluated baseline classifiers, while maintaining strong precision, recall, and F1-score across both classes.

Table 15 summarizes binary classification results on CICIDS2017. The proposed AFI-ID framework achieves an overall accuracy of 98.33% that is higher than that of LR (88.63%), ET (94.48%), and XGB (95.31%).

**Table 15:** Binary classification performance on CICIDS2017.

Classifier	Class	Precision	Recall	F1 score	Accuracy (%)
Logistic regression	Normal	0.9381	0.9191	0.9285	88.63
	Intrusion	0.695	0.7525	0.7226	
Extra trees	Normal	0.9653	0.9659	0.9656	94.48
	Intrusion	0.8607	0.8584	0.8595	
XGB	Normal	0.9922	0.949	0.9701	95.31
	Intrusion	0.8234	0.9696	0.8905	
AFI-ID	Normal	0.9947	0.9845	0.9896	98.33
	Intrusion	0.9391	0.9787	0.9585	

In the case of the Normal class, the precision of AFI-ID is 0.9947 and a recall is 0.9845 (F1 = 0.9896). In the case of the Intrusion class, the precision is 0.9391, and the recall is 0.9787 (F1 = 0.9585). AFI-ID also has a better intrusion recall (0.9787 vs. 0.9696) when compared to XGB, yet it also has high precision, thus, a more balanced precision-recall trade-off.

The ROC curves shown in Figure 11 consistently demonstrate the superiority of AFI-ID. The AUC of AFI-ID is 0.9976, which is high when compared to XGB (0.9923), ET (0.9704), and LR (0.8891). The high global separability is verified by almost unity of the AUC in the case of high-class imbalance. The overall binary classification performance of AFI-ID on UNSW-NB15 and CICIDS2017, including class-imbalance-sensitive metrics, is summarized in Table 16.

**Figure 11:**
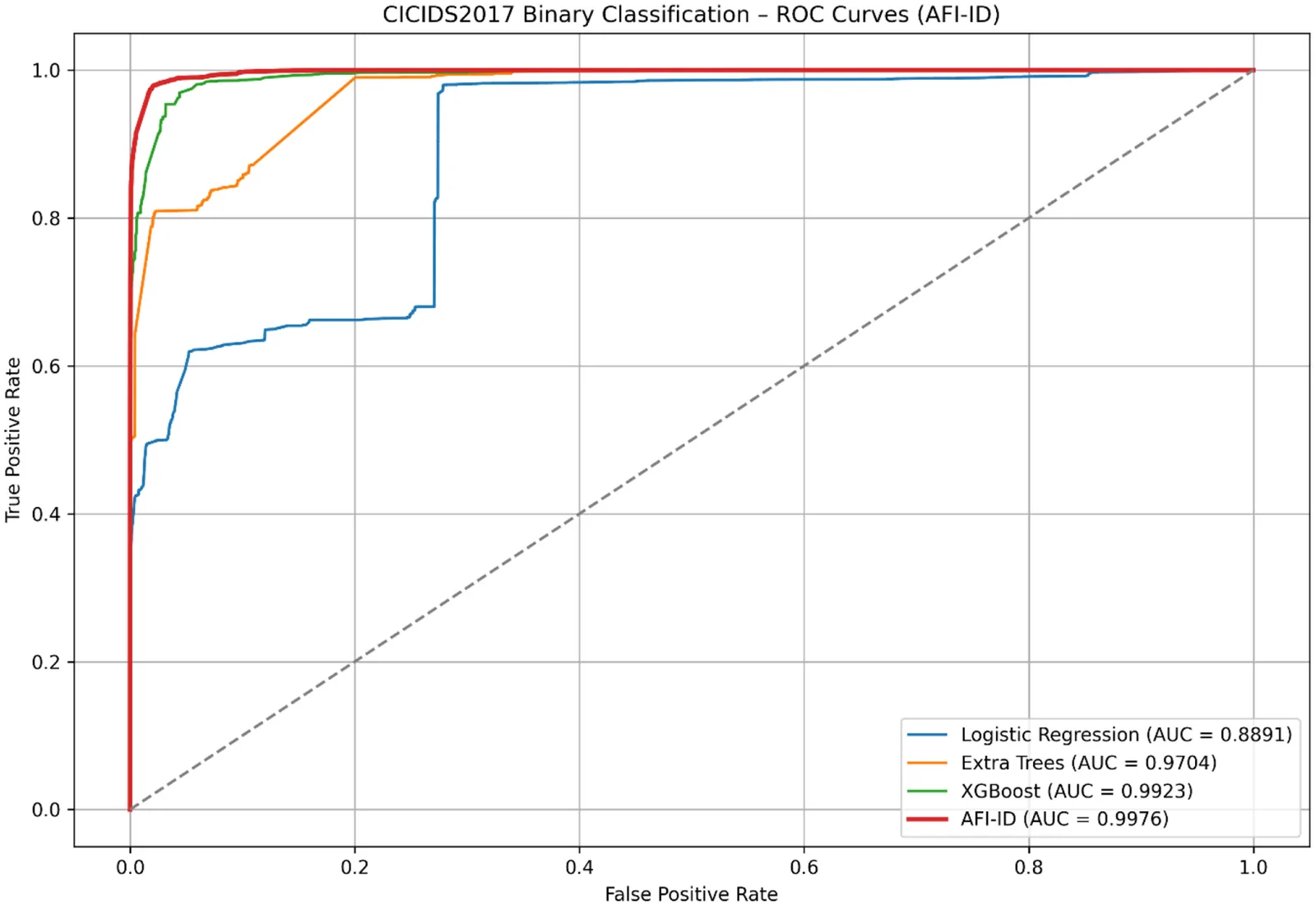
ROC curve-CICIDS2017 (binary classification).

**Table 16:** Overall binary classification metrics.

Dataset	Accuracy (%)	Macro-F1	Balanced accuracy	MCC	ROC-AUC	PR-AUC
UNSW-NB15	93.31	0.9273	0.9323	0.8554	0.9851	0.9916
CICIDS2017	98.33	0.9691	0.9808	0.9389	0.9980	0.9917

The misclassification is also minimized as observed in the confusion matrices of Figure 12. AFI-ID reduces false positives, compared to LR and ET, as well as false negatives, when compared to XGB. The key to this balanced improvement is especially in a large-scale network setting where operational costs are both associated with a false alarm and a missed intrusion. This reduction in misclassification highlights the effectiveness of attention-based aggregation in improving both detection sensitivity and false alarm control.

**Figure 12:**
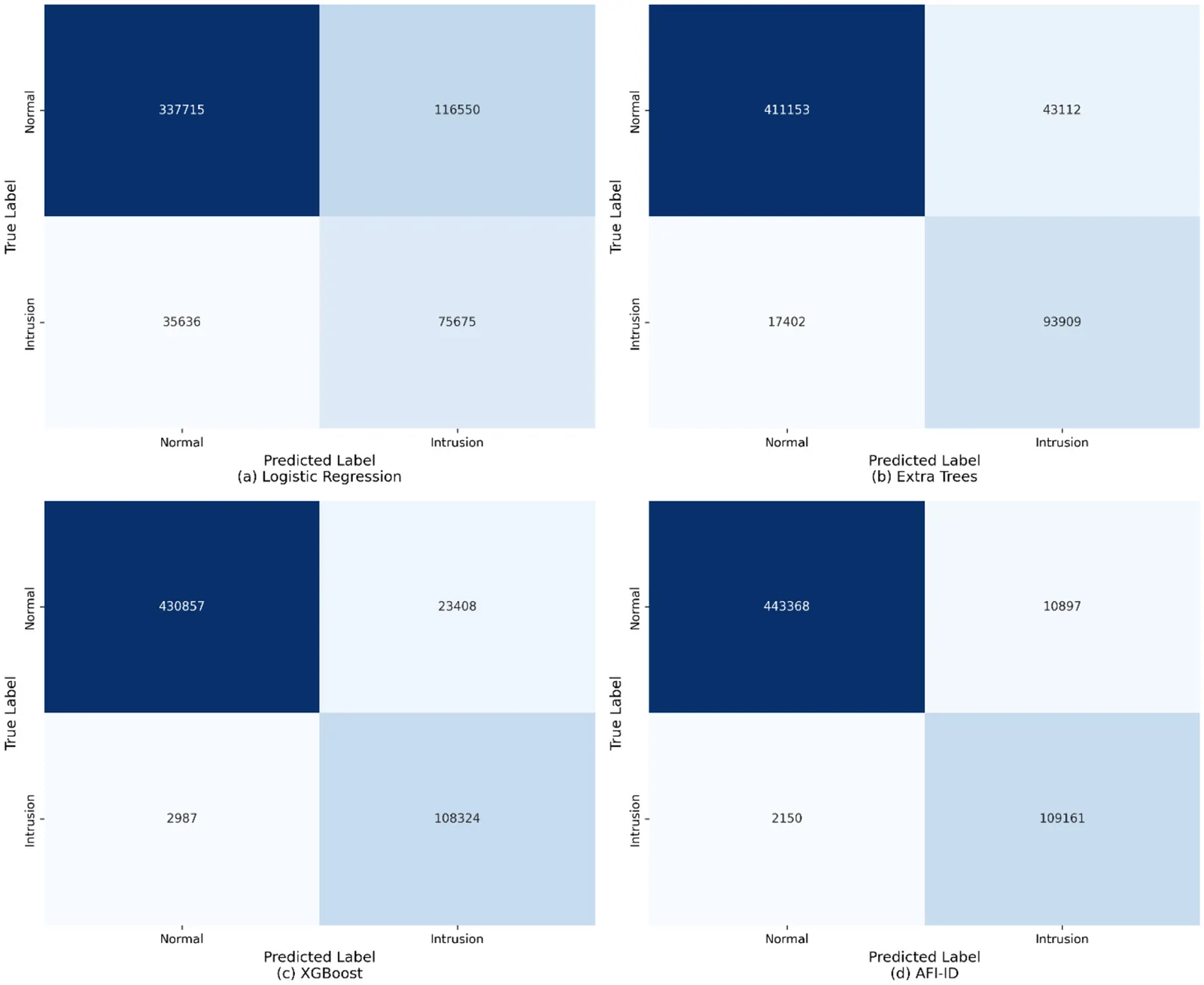
Performance comparison of the base classifiers and AFI-ID: **(a)** Logistic Regression, **(b)** Extra Trees, **(c)** XGBoost, and **(d)** AFI-ID.

The performance gains on CICIDS2017 indicate that AFI-ID scales effectively to large and heterogeneous traffic environments. While binary classification focuses on coarse-grained discrimination, fine-grained multiclass differentiation is required in actual intrusion detection. Multiclass performance is analyzed in the next subsection.

Overall, AFI-ID demonstrates strong aggregate binary detection performance on CICIDS2017. However, the multiclass results show that performance is not uniformly established across all attack categories, particularly for extremely rare classes with very limited test-set support. Therefore, the multiclass findings should be interpreted primarily in terms of aggregate performance and the measurable performance of adequately represented classes, while rare-class generalization remains a limitation.

### 5.7 Multiclass detection

Multiclass intrusion detection presents substantially higher complexity than binary classification due to overlapping attack patterns and severe class imbalance. Here, AFI-ID is compared with LR, ET, and XGB on UNSW-NB15 and CICIDS2017 using Accuracy, Macro-F1, Balanced Accuracy, MCC, ROC-AUC, PR-AUC, and per-class Precision, Recall, and F1-score, together with confusion-matrix and ROC analyses. Macro-averaged metrics are emphasized because they assign equal importance to each attack category and are therefore more informative under the severe class imbalance present in the datasets.

Table 17 shows the multiclass performance of UNSW-NB15. LR attains a total accuracy of 62.04%, which is high for the dominant classes like Generic (F1 = 0.9829) and Normal (F1 = 0.8072). The minority categories of Worms (F1 = 0.0245), Shellcode (F1 = 0.1260) and Analysis (F1 = 0.0903) have significantly lower performance, which indicates poor minority-class generalization.

**Table 17:** Multiclass classification performance on UNSW-NB15.

Classifier	Class	Precision	Recall	F1-score	Accuracy (%)
Logistic regression	Analysis	0.0558	0.2374	0.0903	62.04
	Backdoor	0.0535	0.3927	0.0942	
	DoS	0.3025	0.2100	0.2479	
	Exploits	0.7487	0.3964	0.5184	
	Fuzzers	0.4420	0.583	0.5028	
	Generic	0.9951	0.971	0.9829	
	Normal	0.9918	0.6806	0.8072	
	Reconnaissance	0.1009	0.0947	0.0977	
	Shellcode	0.0686	0.7748	0.126	
	Worms	0.0125	0.7714	0.0245	
Extra trees	Analysis	0.1235	0.2168	0.1574	71.24
	Backdoor	0.0773	0.2146	0.1137	
	DoS	0.3463	0.6041	0.4402	
	Exploits	0.8457	0.4486	0.5862	
	Fuzzers	0.4603	0.7847	0.5802	
	Generic	0.9997	0.9732	0.9863	
	Normal	0.9969	0.7238	0.8386	
	Reconnaissance	0.703	0.544	0.6133	
	Shellcode	0.1145	0.8146	0.2008	
	Worms	0.0226	0.8571	0.0441	
XGB	Analysis	0.1386	0.2093	0.1668	76.54
	Backdoor	0.1294	0.2811	0.1773	
	DoS	0.3501	0.7166	0.4704	
	Exploits	0.8262	0.4862	0.6122	
	Fuzzers	0.5413	0.7088	0.6139	
	Generic	1.0000	0.9732	0.9864	
	Normal	0.9575	0.8096	0.8774	
	Reconnaissance	0.8454	0.8109	0.8278	
	Shellcode	0.2263	0.9106	0.3626	
	Worms	0.0814	0.8	0.1478	
AFI-ID	Analysis	0.1571	0.1121	0.1309	79.95
	Backdoor	0.1219	0.1266	0.1242	
	DoS	0.3503	0.7242	0.4722	
	Exploits	0.7765	0.5592	0.6502	
	Fuzzers	0.6373	0.6494	0.6433	
	Generic	0.9876	0.9795	0.9835	
	Normal	0.9359	0.8937	0.9143	
	Reconnaissance	0.8586	0.7881	0.8218	
	Shellcode	0.4327	0.6921	0.5325	
	Worms	0.2581	0.4571	0.3299	

ET yields better accuracy of 71.24%, and better discrimination on DoS (F1 = 0.4402), Fuzzers (F1 = 0.5802), and Reconnaissance (F1 = 0.6133). Nonetheless, the weakest detected categories remain Worms (F1 = 0.0441) and Shellcode (F1 = 0.2008).

XGB further improves the accuracy to 76.54%. Reconnaissance (F1 = 0.8278) and Shellcode (F1 = 0.3626) are better in terms of performance, whereas Generic (F1 = 0.9864) and Normal (F1 = 0.8774) demonstrate strong discriminative performance. However, Worms (F1 = 0.1478) and Backdoor (F1 = 0.1773) still exhibit limited detection capability under severe class imbalance conditions.

The proposed AFI-ID framework achieves the highest overall accuracy of 79.95%. Shellcode reaches an F1-score of 0.5325, and Worms achieves an F1-score of 0.3299, representing higher class-level F1-scores than those obtained by the evaluated baseline classifiers. Backdoor and Analysis also demonstrate measurable performance improvement. Notably, the performance of dominant classes like Generic (F1 = 0.9835) and Normal (F1 = 0.9143) do not decrease. These results indicate that improvements in minority-class detection are achieved without substantial degradation in majority-class performance.

Figure 13 presents the confusion matrices for the UNSW-NB15 dataset across the evaluated models. Logistic Regression exhibits significant off-diagonal dispersion, particularly for minority classes such as Shellcode, Worms, and Analysis, indicating high misclassification rates and limited capability in capturing complex class boundaries.

**Figure 13:**
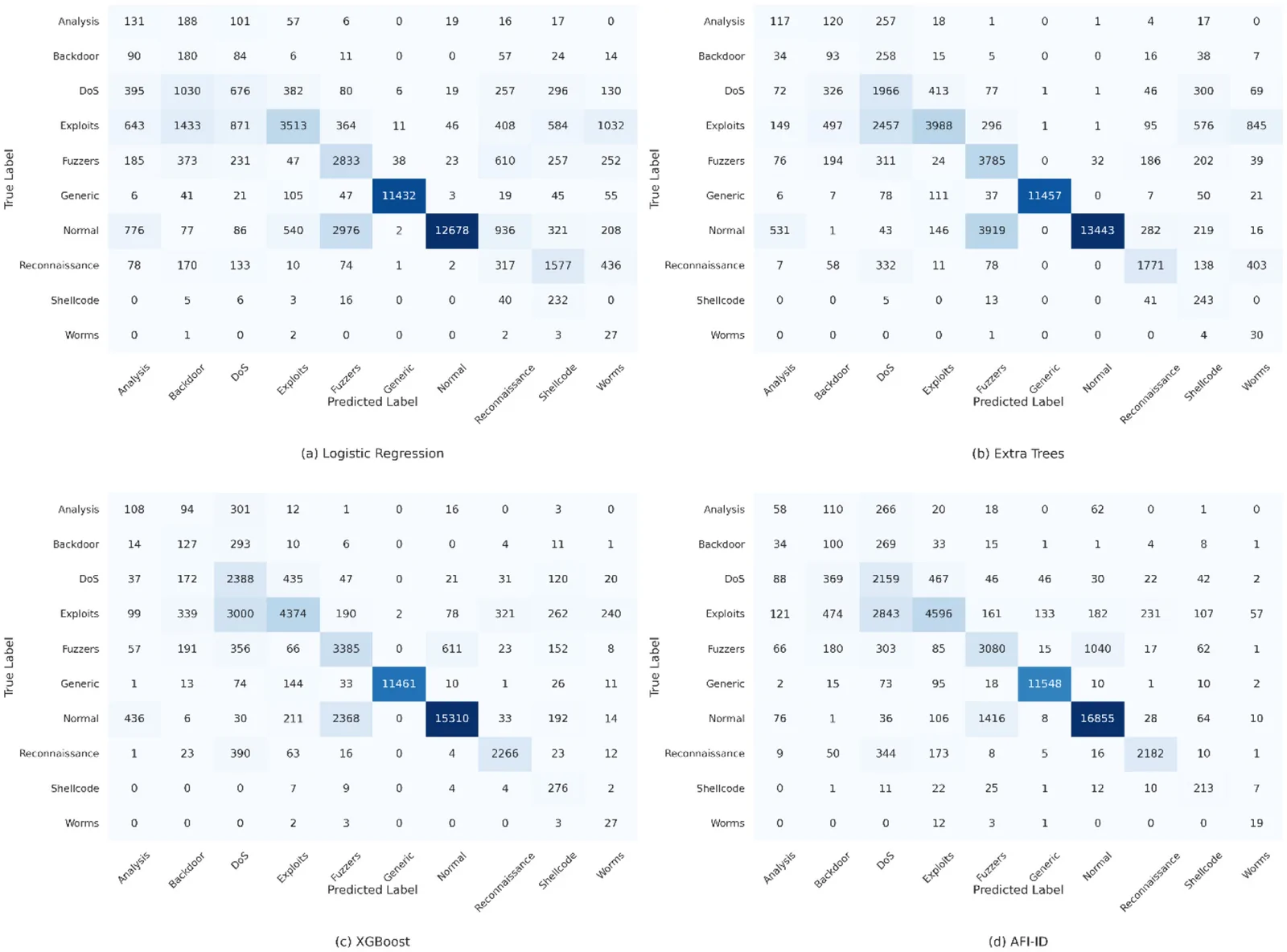
Performance comparison of the base classifiers and AFI-ID: **(a)** Logistic Regression, **(b)** Extra Trees, **(c)** XGBoost, and **(d)** AFI-ID.

Extra Trees reduces confusion among major attack categories, including DoS and Fuzzers; however, notable overlap persists between structurally similar classes such as Exploits and Backdoor, reflecting limited inter-class discrimination.

XGBoost further improves classification performance by enhancing diagonal concentration, particularly for Reconnaissance and Normal traffic, and reducing misclassification across several medium-frequency classes.

In contrast, the proposed AFI-ID framework demonstrates a more distinct diagonal structure across the evaluated classes, including minority categories such as Shellcode and Worms. This indicates improved class-wise separability under the evaluated experimental setting. Overall, the confusion matrices suggest reduced misclassification for several challenging attack categories relative to the baseline classifiers.

As illustrated in Figure 14, the per-class ROC analysis demonstrates that AFI-ID achieves strong discriminative capability across both dominant and minority attack categories. Classes such as Generic, Normal, and Reconnaissance exhibit high AUC values, while minority categories including Worms and Backdoor exhibit comparatively lower discriminative performance under the severe class imbalance. These class-level AUC estimates should be interpreted in conjunction with their available test-set support.

**Figure 14:**
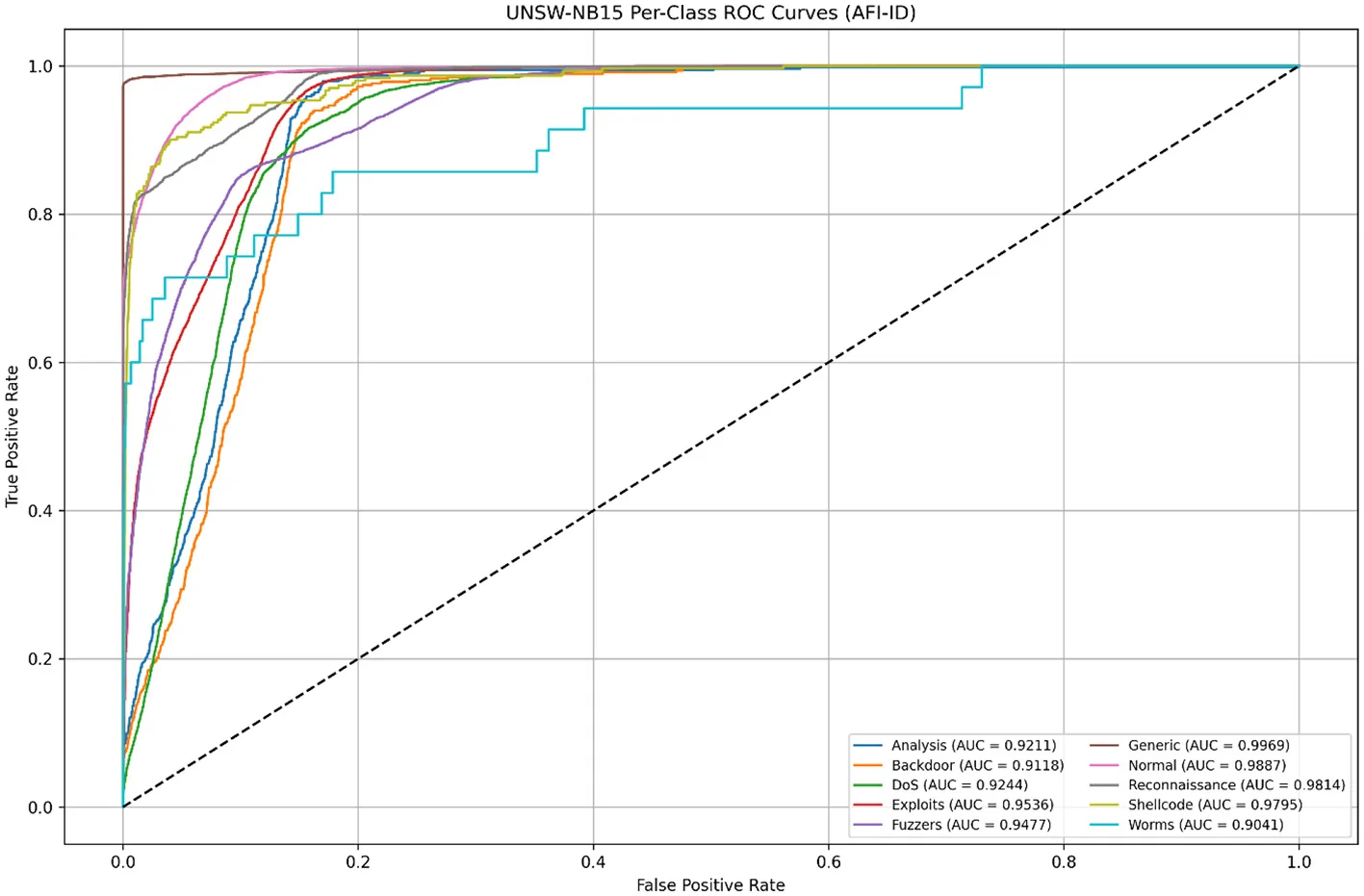
Per-Class ROC Curves for AFI-ID on UNSW-NB15 (multiclass classification).

The micro-averaged ROC-AUC provides an aggregate measure of ranking discrimination across all instances, but it can be influenced strongly by the dominant classes. Therefore, Macro-F1, Balanced Accuracy, MCC, and macro PR-AUC are also reported to provide a more class-balanced assessment of multiclass detection performance.

Table 18 provides an overview of the multiclass results for CICIDS2017. Logistic Regression achieves an overall accuracy of 71.91%, but exhibits weak performance for several minority attack categories, including FTP-Patator, Infiltration, and the Web Attack subclasses. Extra Trees improves the overall accuracy to 81.41% and provides strong F1-scores for DDoS (0.9877), DoS GoldenEye (0.9287), and DoS Slowhttptest (0.9356); however, minority categories such as Bot and SSH-Patator remain difficult to classify. XGBoost further improves the overall accuracy to 90.97%, with high F1-scores for DDoS (0.9947), PortScan (0.9967), and FTP-Patator (0.9785), while Web Attack subclasses and Infiltration remain challenging.

**Table 18:** Multiclass classification performance on CICIDS2017.

Classifier	Class	Precision	Recall	F1 score	Accuracy (%)
Logistic regression	BENIGN	0.9317	0.7232	0.8143	71.91
	Bot	0.0145	0.0513	0.0226	
	DDoS	0.7453	0.552	0.6342	
	DoS GoldenEye	0.1107	0.6942	0.1909	
	DoS Hulk	0.7319	0.6318	0.6782	
	DoS Slowhttptest	0.0797	0.8455	0.1457	
	DoS slowloris	0.0311	0.2069	0.0541	
	FTP-Patator	0	0	0	
	Infiltration	0	0	0	
	PortScan	0.6113	0.9969	0.7579	
	SSH-Patator	0.0592	0.5085	0.1061	
	Web Attack—Brute Force	0	0	0	
	Web Attack—Sql Injection	0	0	0	
	Web Attack—XSS	0.0092	1	0.0181	
Extra trees	BENIGN	0.9996	0.7986	0.8878	81.41
	Bot	0.0205	0.9744	0.0402	
	DDoS	0.9841	0.9914	0.9877	
	DoS GoldenEye	0.8821	0.9806	0.9287	
	DoS Hulk	0.965	0.7182	0.8235	
	DoS Slowhttptest	0.8862	0.9909	0.9356	
	DoS slowloris	0.9545	0.9052	0.9292	
	FTP-Patator	0.1077	1	0.1945	
	Infiltration	0	0	0	
	PortScan	0.8279	0.9994	0.9056	
	SSH-Patator	0.0188	0.9915	0.0369	
	Web Attack—Brute Force	0.0599	0.3333	0.1015	
	Web Attack—Sql Injection	0	0	0	
	Web Attack—XSS	0.0478	0.8462	0.0905	
XGB	BENIGN	0.9995	0.8888	0.9409	90.97
	Bot	0.0334	1	0.0647	
	DDoS	0.9918	0.9977	0.9947	
	DoS GoldenEye	0.7348	0.9951	0.8454	
	DoS Hulk	0.7771	0.9952	0.8727	
	DoS Slowhttptest	0.6832	1	0.8118	
	DoS slowloris	0.7055	0.9914	0.8244	
	FTP-Patator	0.9578	1	0.9785	
	Infiltration	0	0	0	
	PortScan	0.994	0.9994	0.9967	
	SSH-Patator	0.0549	0.9661	0.1039	
	Web Attack—Brute Force	0.0532	0.5	0.0962	
	Web Attack—Sql Injection	0	0	0	
	Web Attack—XSS	0.039	0.6154	0.0734	
AFI-ID	BENIGN	0.9981	0.9219	0.9585	93.54
	Bot	0.0353	0.7692	0.0675	
	DDoS	0.9946	0.9988	0.9967	
	DoS GoldenEye	0.75	0.9612	0.8426	
	DoS Hulk	0.8489	0.9896	0.9138	
	DoS Slowhttptest	0.9464	0.9636	0.955	
	DoS slowloris	0.8321	0.9828	0.9012	
	FTP-Patator	0.9693	0.9937	0.9814	
	Infiltration	0	0	0	
	PortScan	0.9944	0.9991	0.9967	
	SSH-Patator	0.0666	0.9322	0.1243	
	Web Attack—Brute Force	0.0568	0.5	0.102	
	Web Attack—Sql Injection	0	0	0	
	Web Attack—XSS	0.2258	0.5385	0.3182	

The proposed AFI-ID framework achieves an overall accuracy of 93.54% in the original multiclass evaluation. It obtains high F1-scores for the dominant and moderately represented categories, including BENIGN (0.9585), DDoS (0.9967), PortScan (0.9967), FTP-Patator (0.9814), DoS Hulk (0.9138), DoS Slowhttptest (0.9550), and DoS slowloris (0.9012). Performance for the rarer categories remains substantially lower, particularly for Bot, SSH-Patator, and the Web Attack subclasses.

The minority-class results require careful interpretation because of the extremely small class support in the evaluated CICIDS2017 sample. Following the stratified sampling procedure, Infiltration and Web Attack–SQL Injection contained only 4 and 2 instances, respectively, while Heartbleed contained only 1 instance and was therefore excluded from the subsequent 14-class experiment. After the subsequent stratified train–validation–test partitioning, only 1 Infiltration instance was present in the test set, whereas Web Attack–SQL Injection had 0 test instances. AFI-ID did not correctly classify the single Infiltration test instance, resulting in an F1-score of 0.0000. The zero-valued metrics for Web Attack–SQL Injection should not be interpreted as evidence of detection failure because this class had no test samples. These extremely small supports substantially limit the reliability of class-specific performance estimates for these categories and prevent the evaluated sample from being considered representative of the full-dataset behavior of these rare attack classes.

#### 5.7.1 Minority-class performance

The class-wise results reveal an important limitation associated with the extremely rare CICIDS2017 attack categories. In the evaluated test partition, Infiltration was represented by only 1 sample, while Web Attack–SQL Injection had no test samples. Consequently, the F1-score for Infiltration was 0, whereas the zero F1-score reported for Web Attack–SQL Injection is not directly evaluable because no test instance was available for this class. These extremely small or absent test supports substantially limit the reliability of class-specific performance estimates and highlight the need for larger and more representative samples when evaluating rare attack categories.

#### 5.7.2 Interpretation of micro-ROC-AUC

The high micro-averaged ROC-AUC should also be interpreted in the context of the severe class imbalance. ROC-AUC measures the ability of the classifier to rank instances across decision thresholds, whereas F1-score evaluates the final class assignments at a particular operating point. Moreover, micro-averaged ROC-AUC aggregates contributions from all instances and is therefore strongly influenced by the dominant classes. Consequently, a high micro-ROC-AUC can coexist with weak or unevaluable performance for extremely rare classes. The micro-ROC-AUC should therefore be interpreted as an aggregate discrimination measure rather than evidence of uniformly strong detection across all attack categories. The micro-averaged ROC curves for the evaluated models are presented in Figure 15.

**Figure 15:**
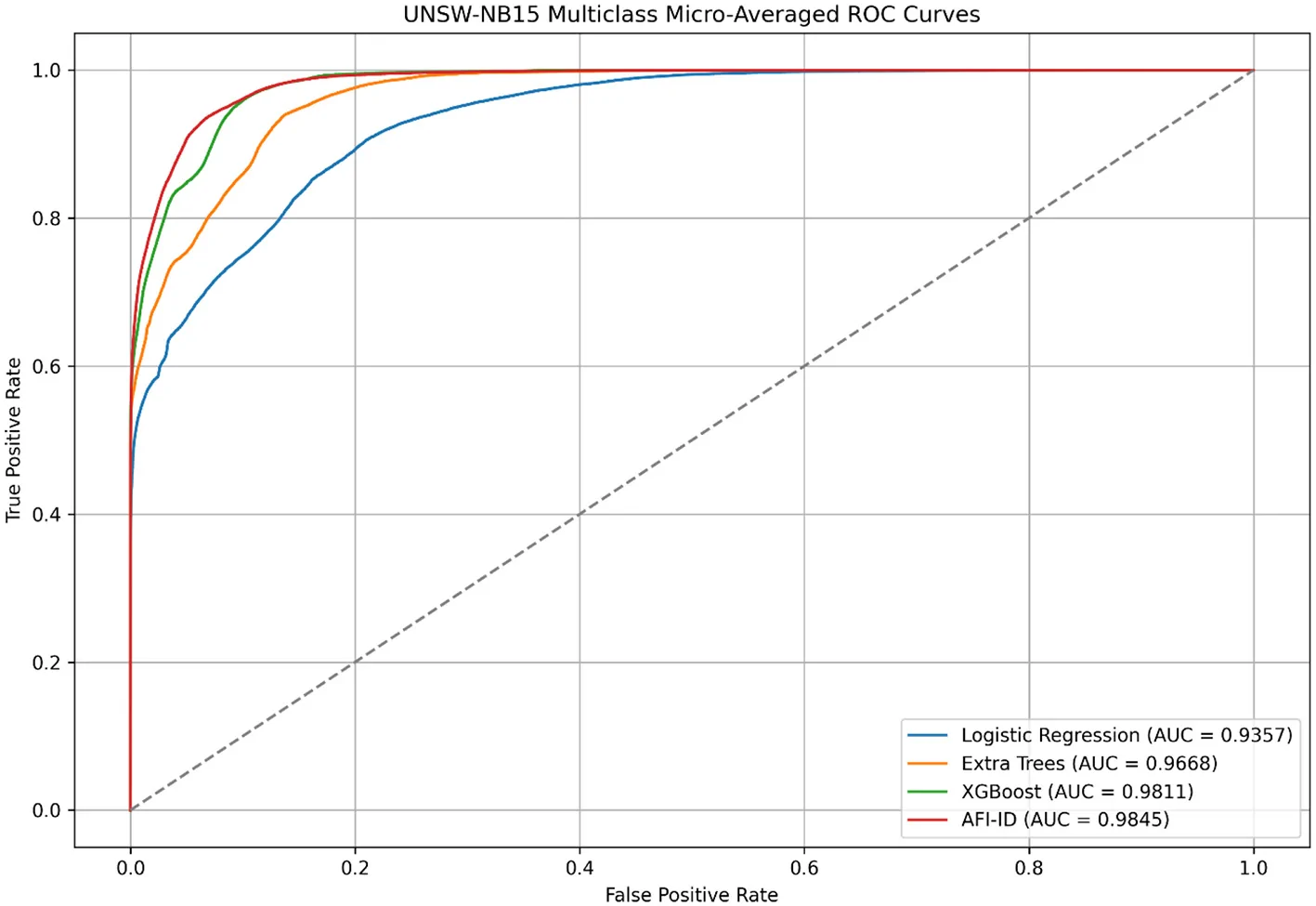
Micro-averaged ROC comparison on UNSW-NB15 (multiclass classification).

The multiclass results on UNSW-NB15 demonstrate the importance of using class-balanced evaluation metrics under the dataset's imbalanced class distribution. AFI-ID achieved an overall accuracy of 79.9583%; however, the Macro-F1 and Balanced Accuracy were 0.5510 and 0.5830, respectively, indicating that aggregate accuracy does not fully represent performance across all attack categories. The MCC of 0.7390 provides additional evidence of a substantial overall association between the predicted and true classes. The Macro ROC-AUC of 0.9492 was lower than the Micro ROC-AUC of 0.9846, while Macro PR-AUC (0.5678) was substantially lower than Micro PR-AUC (0.9089). This difference reflects the influence of the higher-support classes on micro-averaged measures and highlights the importance of macro-averaged metrics for assessing performance across minority attack categories. The per-class results further show substantial variation, with particularly lower F1-scores for Analysis, Backdoor, and Worms, which have relatively limited test-set support. Interpretation of aggregate ROC-AUC and minority-class performance. The revised evaluation also highlights an important distinction between aggregate ranking performance and class-specific classification performance. AFI-ID achieves a micro-averaged ROC-AUC of 0.9992, while its Macro-F1 is 0.6276 and Balanced Accuracy is 0.8004. The high micro-ROC-AUC should therefore not be interpreted as evidence of uniformly strong detection across all attack categories. Micro-averaged ROC-AUC aggregates the contributions of all test instances and is consequently strongly influenced by the substantially larger classes, particularly BENIGN and the major attack categories. In contrast, Macro-F1 gives equal weight to each class and is therefore more sensitive to weak performance in minority categories.

The overall multiclass classification results for UNSW-NB15 and CICIDS2017 are summarized in Table 19. ROC-AUC and F1-score also characterize different aspects of classifier behavior. ROC-AUC measures ranking discrimination across decision thresholds, whereas F1-score evaluates the precision–recall trade-off associated with the final class assignments. Consequently, a classifier may achieve a high aggregate ROC-AUC while still producing weak class-specific F1-scores for rare attack categories. This effect is particularly pronounced for CICIDS2017, where some categories have extremely small test-set support. The micro-ROC-AUC is therefore interpreted as an aggregate discrimination measure rather than evidence of uniformly robust multiclass detection.

**Table 19:** Overall multiclass classification metrics.

Dataset	Accuracy (%)	Macro-F1	Balanced accuracy	MCC	Macro ROC-AUC	Macro PR-AUC	Micro ROC-AUC	Micro PR-AUC
UNSW-NB15	79.9583	0.5510	0.5830	0.7390	0.9492	0.5678	0.9846	0.9089
CICIDS2017	93.5472	0.6276	0.8004	0.8366	0.9423	0.7112	0.9992	0.9912

Formal statistical significance testing was conducted for the repeated UNSW-NB15 binary experiments and for the controlled AFI-ID vs. HAMC-ID comparison. The CICIDS2017 multiclass results are therefore interpreted descriptively rather than as statistically significant differences. Accordingly, the observed multiclass performance differences on CICIDS2017 should not be interpreted as formally established statistical improvements.

Figure 16 presents the confusion matrices for the CICIDS2017 dataset across the evaluated models. The baseline classifiers exhibit strong diagonal dominance for high-frequency classes such as BENIGN and major DoS variants; however, noticeable misclassification persists in minority and structurally similar classes, particularly within Web Attack categories and SSH-Patator.

**Figure 16:**
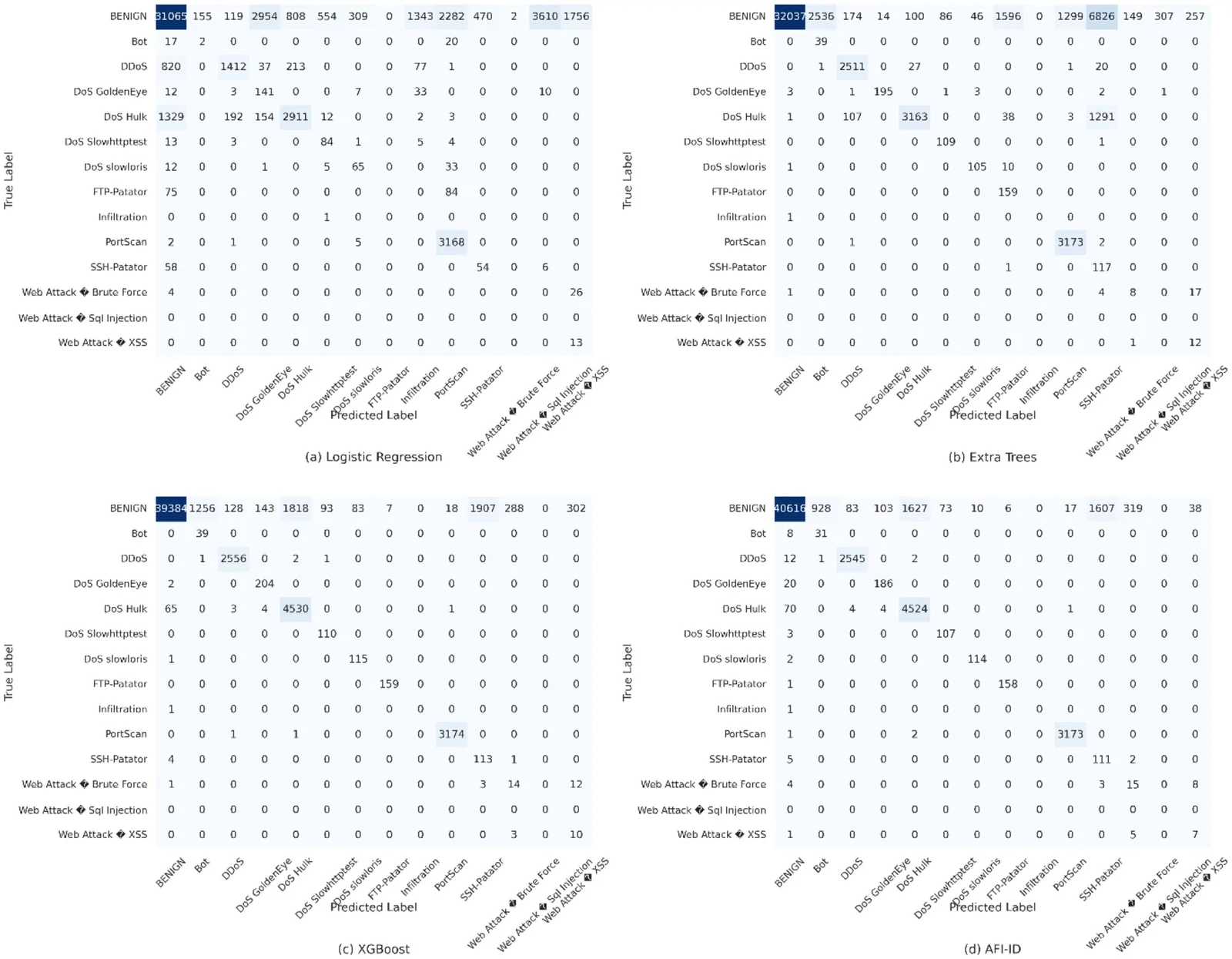
Performance comparison of the base classifiers and AFI-ID: **(a)** Logistic Regression, **(b)** Extra Trees, **(c)** XGBoost, and **(d)** AFI-ID.

In contrast, the proposed AFI-ID framework demonstrates improved diagonal concentration across multiple attack categories, including DoS variants and FTP-Patator. The confusion matrix also shows reduced inter-class confusion for several challenging categories, including Web Attack–XSS, under the evaluated experimental setting.

Overall, the confusion matrices indicate that AFI-ID provides improved class-wise consistency relative to the evaluated baseline classifiers, while extremely rare categories remain difficult to classify reliably.

As illustrated in Figure 17, the per-class ROC analysis further reveals strong separability for major attack categories such as DDoS, PortScan, and FTP-Patator. However, extremely low-frequency classes such as Infiltration and SQL Injection exhibit limited or unreliable class-level discrimination because of their sparse representation. Future work may investigate few-shot, zero-shot, and other data-efficient learning strategies to improve detection of extremely rare attack categories.

**Figure 17:**
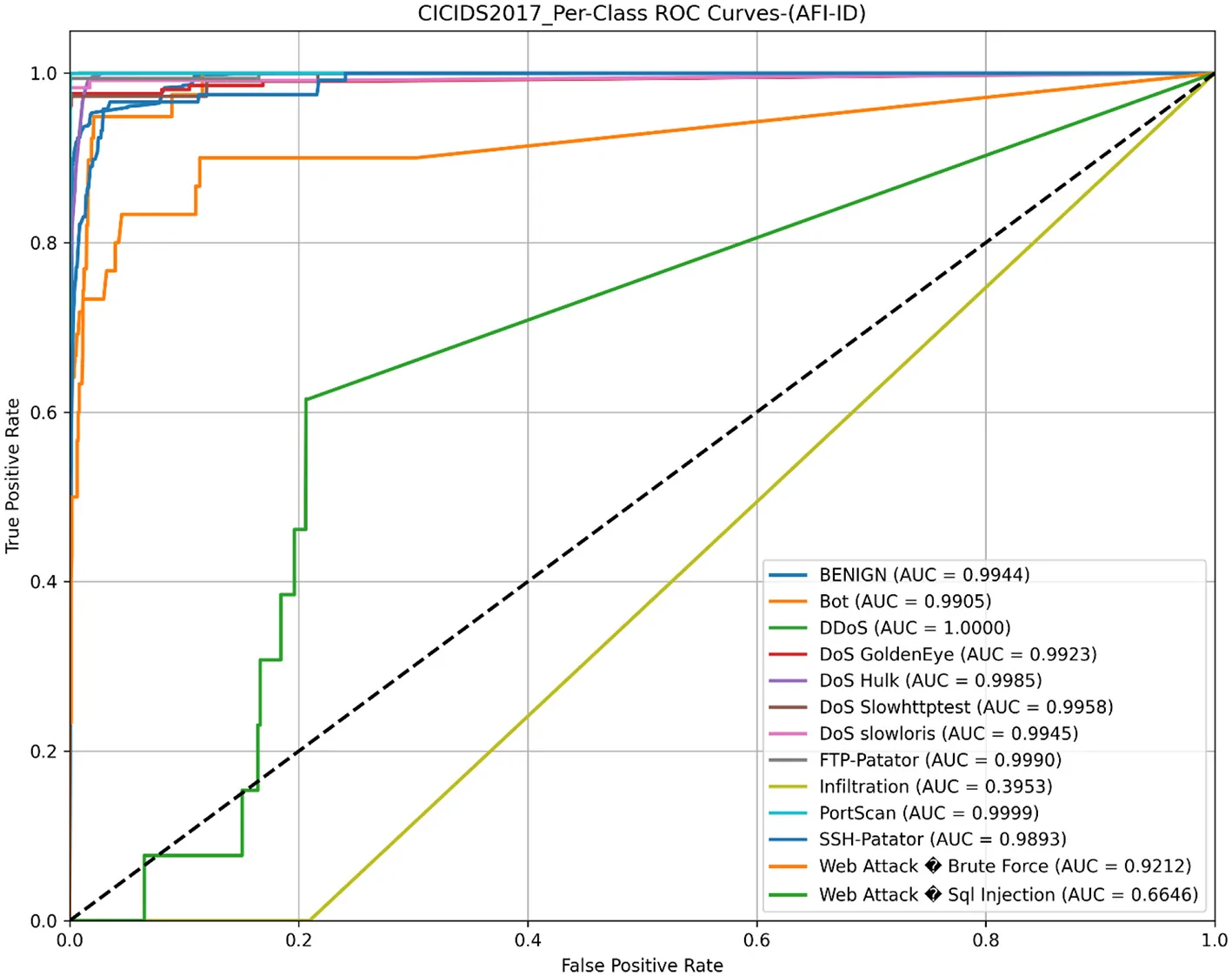
Per-class ROC curves for AFI-ID on CICIDS2017 (multiclass classification).

Figure 18 reveals that multiclass micro-averaged AUC of AFI-ID is 0.999, which is higher than XGB (0.998), ET (0.992), and LR (0.889). It reveals a better overall separability of the attack categories using the one-vs.-rest evaluation framework even with extreme class imbalance.

**Figure 18:**
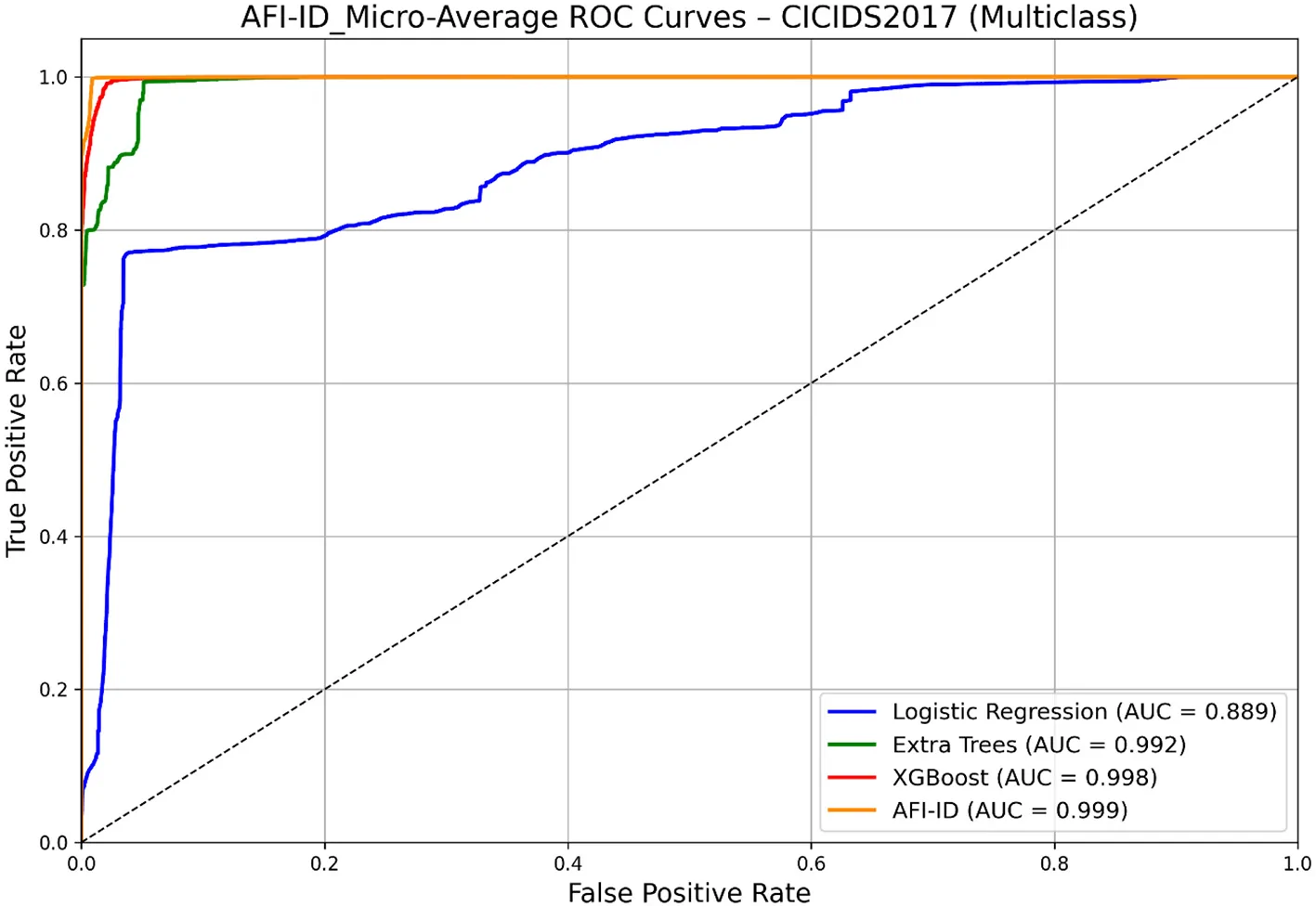
Micro-averaged ROC-CICIDS2017 (multiclass classification).

AFI-ID demonstrates strong aggregate multiclass discrimination under the evaluated CICIDS2017 setting; however, class-wise performance remains uneven for extremely rare attack categories because of their limited representation.

These results demonstrate that AFI-ID improves overall classification performance and enhances detection for several minority classes. However, extremely rare classes such as Infiltration and certain Web Attack categories remain challenging due to severe data scarcity and class imbalance. The limited detection capability for extremely rare classes, such as Infiltration, is primarily due to the very limited number of training samples. In such cases, both base classifiers and the attention-based aggregation mechanism lack sufficient information to learn reliable patterns, highlighting a fundamental limitation of data-driven intrusion detection models under extreme imbalance conditions.

### 5.8 Training dynamics and convergence analysis

While ROC curves and confusion matrices demonstrate the discriminative capability of the proposed AFI-ID framework, it is equally important to analyze its training behavior and convergence characteristics. To this end, learning curves representing training and validation accuracy across epochs are examined for both binary and multiclass scenarios.

As illustrated in Figure 19, the training accuracy increases steadily and converges toward a high value, indicating effective learning of discriminative patterns. The validation accuracy exhibits moderate fluctuations across epochs, which can be attributed to variations in traffic distribution and inherent class imbalance in the UNSW-NB15 dataset. Despite these fluctuations, the validation trend remains stable without significant divergence from the training curve, suggesting that the model does not suffer from severe overfitting and maintains generalization under the evaluated benchmark conditions. The observed difference between training and validation performance remained small throughout experimentation, indicating limited overfitting and stable generalization across unseen data.

**Figure 19:**
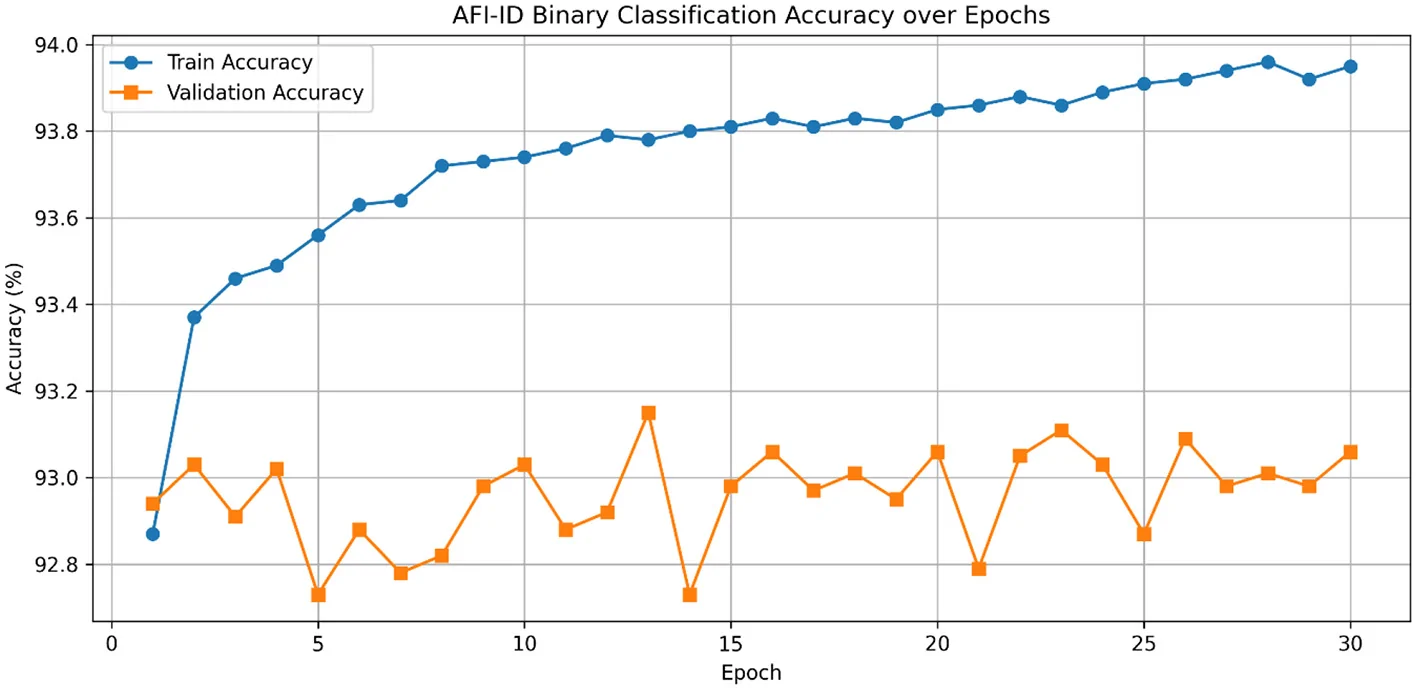
Learning curve of AFI-ID for binary classification on UNSW-NB15.

As shown in Figure 20, the model exhibits consistent convergence behavior under multiclass classification on CICIDS2017. Compared to the binary scenario, the validation accuracy demonstrates higher variability due to increased class complexity, overlapping attack patterns, and severe class imbalance. Nevertheless, the training accuracy converges smoothly, and the validation performance remains within an acceptable range. Similarly, the multiclass learning curves demonstrate that the gap between training and validation performance remains moderate despite increased class complexity, suggesting that the proposed AFI-ID framework maintains robust generalization capability under highly imbalanced conditions. This indicates that AFI-ID effectively captures complex inter-class relationships while preserving generalization performance.

**Figure 20:**
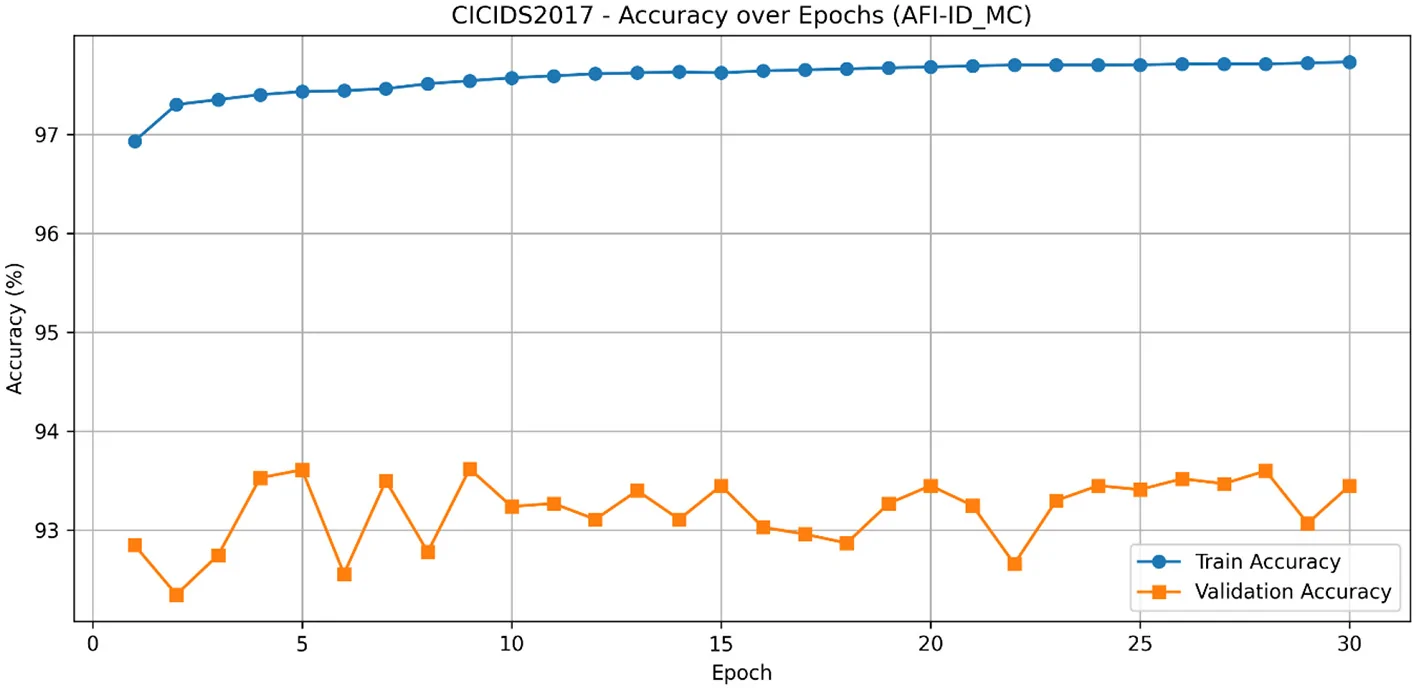
Learning curve of AFI-ID for multiclass classification on CICIDS2017.

Overall, the consistent convergence patterns observed across both datasets confirm the robustness and stability of the proposed AFI-ID model under varying classification complexities.

### 5.9 Comparison with state-of-the-art intrusion detection methods

To further evaluate the positioning of the proposed AFI-ID framework, its performance is compared with recently reported intrusion detection models from the literature. The comparison includes recent IDS approaches, with particular emphasis on methods employing feature-selection or feature-reduction strategies, as these provide a more relevant contextual basis for assessing the adaptive feature intelligence component of AFI-ID. Representative deep learning, ensemble, attention-based, and other recent IDS architectures are also included where comparable results on UNSW-NB15 and CICIDS2017 are available.

A contextual comparison of AFI-ID with recent intrusion detection methods on UNSW-NB15 and CICIDS2017 is presented in Table 20. The literature comparison is presented separately from the controlled experiments conducted in this study. The LR, ET, XGB, HAMC-ID, and AFI-ID results generated using the experimental protocols described in this work constitute controlled same-protocol comparisons. In contrast, results reported by external studies are treated as contextual literature comparisons because differences in data partitions, preprocessing, feature selection, class-balancing procedures, feature representations, and evaluation metrics may affect the reported performance. Therefore, the literature results are used to position AFI-ID within recent IDS research rather than to establish a strictly controlled numerical ranking across studies.

**Table 20:** Contextual comparison with recent intrusion detection methods on UNSW-NB15 and CICIDS2017.

Dataset	Method	Year	Approach	Task	Accuracy (%)	F1-score (%)
UNSW-NB15	Rashid et al., 2022	2022	Tree-based stacking	Binary	92.10	–
	Zhang et al., 2023	2023	BiLSTM + attention	Binary	92.50	–
	Emirmahmutoğlu and Atay (2025)	2025	Feature selection + decision tree	Binary	92.44	95.07
	AFI-ID (proposed)	–	Adaptive feature intelligence + attention-based stacking	Binary	93.31	–
CICIDS2017	Wang et al., 2023	2023	Random forest	Binary	99.80	99.70
	Tripathi et al., 2024	2024	Weighted feature selection + RF	Binary	99.90	–
	Adewole et al., 2025	2025	XGBoost + rule induction	Binary	99.91	99.91
	Jeong et al., 2026	2026	Centralized baseline	Binary	98.84 ± 0.04	98.16 ± 0.06
	AFI-ID (proposed)	–	Adaptive feature intelligence + attention-based stacking	Binary	98.33	–

The comparison includes recent IDS methods, with emphasis on approaches employing feature-selection or feature-reduction strategies. Reported results are taken from the respective studies under their original experimental settings. Differences in pre-processing, data partitioning, feature selection, class balancing, and evaluation protocols make the comparison contextual rather than strictly controlled. AFI-ID applies validation-driven hybrid feature selection before classification, reducing the input feature space while retaining informative attributes. The controlled comparisons within this study remain the primary basis for assessing AFI-ID.

The expanded comparison places AFI-ID within the range of performance reported by the selected recent intrusion detection approaches. However, differences in experimental protocols prevent direct numerical ranking across studies. Recent CNN-, recurrent-, Transformer-, and graph-based IDS architectures provide strong representation-learning capabilities, whereas AFI-ID adopts a different design strategy by combining adaptive feature selection with heterogeneous classifier fusion and an attention-based meta-classifier, thereby reducing the input feature space while retaining complementary classifier information.

Unlike many hybrid feature selection approaches that rely on heuristic feature aggregation, AFI-ID integrates statistical relevance and model-based importance through a validation-driven weighted fusion strategy. At the ensemble level, structured meta-features derived from base learner predictions are processed using an attention-based BiLSTM meta-classifier, enabling context-aware weighting of classifier contributions.

The integrated AFI-ID architecture combines adaptive feature selection, heterogeneous classifier fusion, and attention-based meta-classification while reducing the dimensionality of the input feature space. Moreover, AFI-ID was evaluated on both UNSW-NB15 and CICIDS2017, providing evidence of its applicability across two widely used intrusion detection benchmarks with different traffic characteristics and class distributions.

Overall, the comparison indicates that the integration of adaptive feature intelligence with attention-based ensemble aggregation provides a competitive combination of feature reduction and classification performance under the evaluated experimental conditions. The controlled same-protocol experiments provide the primary evidence for improvements over the baseline classifiers, while the expanded literature comparison positions AFI-ID relative to recent IDS approaches without assuming direct equivalence across different experimental protocols.

The observed performance of AFI-ID across the evaluated settings can be interpreted in terms of the complementary contributions of its feature-selection and ensemble-learning components. The hybrid adaptive feature intelligence module refines the feature space by combining statistical dependency measures with model-driven importance, resulting in a compact subset of informative features and reducing the influence of potentially redundant attributes.

At the ensemble level, the attention-based meta-classifier assigns attention-guided importance weights to base learner outputs based on input characteristics, enabling attention-guided decision fusion. The attention mechanism enables adaptive weighting of the base-learner outputs according to the learned meta-feature representation. The resulting contribution to minority-class detection is therefore evaluated through the per-class Precision, Recall, and F1 results rather than inferred solely from aggregate performance metrics.

The reduced feature dimensionality also decreases the number of input attributes processed by the downstream classifiers. Together, adaptive feature selection and attention-based meta-classification provide a coherent explanation for the observed performance and compact feature representation of AFI-ID under the evaluated experimental conditions.

These results position AFI-ID as an alternative architectural strategy that combines heterogeneous machine-learning classifiers with attention-based meta-classification and adaptive feature selection. AFI-ID provides detection performance with reduced feature dimensionality and attention-guided decision fusion, offering a design that considers both classification performance and feature-space compactness. Recent studies, including weighted feature selection frameworks (Tripathi et al., 2024), hybrid feature selection and multi-stage IDS models (Logeswari et al., 2025b), and ensemble-based intrusion detection systems (Doost et al., 2025), further confirm the growing importance of adaptive feature intelligence and ensemble aggregation strategies in modern intrusion detection research.

The findings indicate that the integration of adaptive feature intelligence with attention-based ensemble aggregation provides a balanced design in terms of detection performance and feature-space compactness under the evaluated experimental conditions.

Recent graph-based intrusion detection approaches have demonstrated the ability to model structural relationships among network entities through graph representations. These approaches may introduce additional graph-construction and computational requirements depending on the underlying implementation. AFI-ID adopts a flow-level tabular representation and instead focuses on adaptive feature intelligence and attention-guided classifier fusion. Thus, the two approaches represent different architectural strategies rather than directly comparable alternatives.

## 6 Ablation study

To assess the contribution and stability of the individual AFI-ID components, the ablation experiments were repeated using five random seeds (7, 21, 42, 84, and 123). Table 21 reports the resulting mean ± standard deviation across the five runs. The complete AFI-ID configuration achieved an accuracy of 93.27 ± 0.09%, Macro-F1 of 92.71 ± 0.11%, Balanced Accuracy of 92.77 ± 0.18%, MCC of 0.8543 ± 0.0022, ROC-AUC of 0.9860 ± 0.0006, and PR-AUC of 0.9922 ± 0.0004. Removing the filtration component reduced accuracy to 92.80 ± 0.20% and MCC to 0.8435 ± 0.0046, indicating the contribution of the statistical relevance component. Removing the embedding component also resulted in a modest reduction in performance. The all-original-features configuration produced comparable aggregate performance but retained all 42 available features, whereas the PCA-based configuration resulted in the lowest performance among the evaluated variants. Overall, the relatively small standard deviations across the five seeds indicate that the ablation results are stable under the tested random initializations. The results are therefore interpreted as evidence of component-level contribution and performance stability rather than as uniformly large improvements over every ablation variant.

**Table 21:** Five-seed ablation analysis of AFI-ID on UNSW-NB15. Results are reported as mean ± standard deviation across five random seeds.

Ablation variant	Features	Accuracy (%)	Macro-F1 (%)	Balanced accuracy (%)	MCC	ROC-AUC	PR-AUC
AFI-ID (full)	19.0 ± 0.7	93.27 ± 0.09	92.71 ± 0.11	92.77 ± 0.18	0.8543 ± 0.0022	0.9860 ± 0.0006	0.9922 ± 0.0004
Without filtration	10.6 ± 0.5	92.80 ± 0.20	92.17 ± 0.23	92.04 ± 0.37	0.8435 ± 0.0046	0.9841 ± 0.0007	0.9910 ± 0.0005
Without embedding	36.0 ± 0.0	93.13 ± 0.14	92.58 ± 0.15	92.75 ± 0.17	0.8517 ± 0.0031	0.9854 ± 0.0004	0.9919 ± 0.0003
Without adaptive threshold	21.0 ± 0.0	93.25 ± 0.08	92.71 ± 0.07	92.84 ± 0.05	0.8542 ± 0.0013	0.9858 ± 0.0003	0.9921 ± 0.0002
All original features	42.0 ± 0.0	93.25 ± 0.13	92.72 ± 0.13	92.93 ± 0.14	0.8545 ± 0.0026	0.9858 ± 0.0004	0.9921 ± 0.0003
PCA-based	21.0 ± 0.0	91.99 ± 0.27	91.28 ± 0.28	91.13 ± 0.25	0.8258 ± 0.0057	0.9802 ± 0.0013	0.9887 ± 0.0008

### 6.1 OOF stacking, classifier-order, and meta-learner analysis

To verify that the stacking procedure does not introduce information leakage, a separate 5-fold OOF experiment was conducted using the final 17-feature AFI-ID subset selected under the primary experimental configuration. The training partition contained 154,603 instances and was divided into five stratified folds. For each fold, LR, ET, and XGB were trained using only the remaining 4 folds, with SMOTE applied exclusively to the corresponding fold-training data. OOF predictions were generated for every training instance, and the OOF coverage verification confirmed zero missing predictions for all three base classifiers. The validation and test predictions were subsequently generated using models trained on the complete training partition.

The classifier-order experiment produced closely clustered results across all six permutations. The XGB → LR → ET ordering yielded the highest Accuracy (0.933657), Macro-F1 (0.928385), Balanced Accuracy (0.930158), and MCC (0.856871), while ROC-AUC and PR-AUC remained above 0.986 and 0.992, respectively, for all permutations. The relatively small differences among the six configurations indicate that the stacking performance is not strongly dependent on classifier ordering. Therefore, the results do not support the claim that a particular ordering is intrinsically optimal; rather, they demonstrate that the meta-learner is reasonably robust to permutation of the three classifier representations.

To further evaluate the suitability and stability of the BiLSTM-based aggregation architecture, the meta-classifier comparison was repeated across five random seeds (7, 21, 42, 84, and 123) using the same leakage-free OOF meta-features and experimental protocol. The proposed BiLSTM + Attention model was compared with a conventional MLP meta-classifier and an attention-pooling alternative. Table 22 reports the mean ± standard deviation across the five runs.

**Table 22:** Five-seed comparison of alternative meta-classifier aggregation strategies.

Meta-classifier	Accuracy (%)	Macro-F1 (%)	Balanced accuracy (%)	MCC	ROC-AUC	PR-AUC
BiLSTM + attention	94.5614 ± 0.0832	94.1214 ± 0.0964	94.2445 ± 0.1505	0.8825 ± 0.0020	0.9907 ± 0.0002	0.9949 ± 0.0001
MLP	94.5501 ± 0.0552	94.1132 ± 0.0628	94.2664 ± 0.1188	0.8824 ± 0.0013	0.9906 ± 0.0002	0.9947 ± 0.0001
Attention pooling	94.1632 ± 0.1133	93.6568 ± 0.1261	93.5426 ± 0.1944	0.8732 ± 0.0025	0.9861 ± 0.0015	0.9882 ± 0.0022

Values are reported as mean ± standard deviation across five random seeds (7, 21, 42, 84, and 123).

To further evaluate the suitability of the BiLSTM-based aggregation architecture, a meta-classifier comparison was conducted using the same leakage-free OOF meta-features and training, validation, and testing protocol. The proposed BiLSTM + Attention model was compared with a conventional MLP meta-classifier and an attention-pooling alternative. Each configuration was evaluated using five random seeds (7, 21, 42, 84, and 123), and the results are reported as mean ± standard deviation in Table 23. The BiLSTM + Attention model achieved the highest mean Accuracy (94.5614%), Macro-F1 (94.1214%), MCC (0.8825), ROC-AUC (0.9907), and PR-AUC (0.9949). The MLP produced a marginally higher Balanced Accuracy (94.2664%) than the BiLSTM + Attention model (94.2445%), while maintaining very similar performance across the other metrics. In contrast, the attention-pooling alternative showed consistently lower performance, with a mean Accuracy of 94.1632%, Macro-F1 of 93.6568%, and MCC of 0.8732. These results indicate that the proposed BiLSTM + Attention aggregation is competitive with a simpler MLP while providing the strongest overall performance across most evaluated metrics. The relatively small difference between BiLSTM + Attention and MLP also indicates that the benefit of the proposed aggregation mechanism should not be interpreted as a large performance gain.

**Table 23:** Classifier-order permutation analysis using leakage-free OOF stacking.

Classifier order	Accuracy	Macro-F1	Balanced accuracy	MCC	ROC-AUC	PR-AUC
LR → ET → XGB	0.9320	0.9264	0.9266	0.8528	0.9863	0.9924
LR → XGB → ET	0.9325	0.9272	0.9298	0.8547	0.9861	0.9923
ET → LR → XGB	0.9331	0.9278	0.9295	0.8558	0.9865	0.9925
ET → XGB → LR	0.9328	0.9272	0.9279	0.8546	0.9858	0.9921
XGB → LR → ET	0.9336	0.9283	0.9301	0.8568	0.9864	0.9925
XGB → ET → LR	0.9327	0.9272	0.9277	0.8544	0.9859	0.9922

To further evaluate the incremental contribution of the proposed AFI-ID framework, an additional comparative experiment was conducted against the previously proposed HAMC-ID model using five random seeds (7, 21, 42, 84, and 123). For each seed, both models were evaluated using identical stratified train–validation–test splits, with the same test instances used for direct comparison. Both models employed the same base learners, attention-based BiLSTM meta-classifier, preprocessing strategy, and SMOTE-based imbalance handling. This controlled experimental setup isolates the contribution of the feature-intelligence mechanism. The primary architectural difference is that AFI-ID replaces the Chi-square and PCA-based feature processing of HAMC-ID with the proposed hybrid adaptive feature-intelligence strategy. In this controlled comparison, AFI-ID used its selected feature representation, whereas HAMC-ID used its corresponding Chi-square-selected and PCA-reduced representation, with all other major experimental components kept consistent.

As shown in Table 22, AFI-ID consistently achieved higher predictive performance than HAMC-ID across the five random seeds under identical train–validation–test splits. AFI-ID obtained a mean accuracy of 92.70 ± 0.18%, F1-score of 94.17 ± 0.15%, recall of 92.26 ± 0.33%, MCC of 0.8455 ± 0.0036, and Kappa of 0.8442 ± 0.0037, compared with 89.57 ± 0.23%, 91.60 ± 0.24%, 88.99 ± 0.87%, 0.7811 ± 0.0028, and 0.7787 ± 0.0038, respectively, for HAMC-ID. AFI-ID also achieved a lower mean log loss of 0.1514 ± 0.0029 compared with 0.1889 ± 0.0038 for HAMC-ID. The relatively small standard deviations across the five seeds indicate stable performance under the tested random initializations. AFI-ID also required less mean training time than HAMC-ID, while the per-sample inference times were comparable.

For an instance-level paired comparison, a representative controlled split with identical test instances was additionally analyzed using McNemar's exact test and paired bootstrap resampling. McNemar's exact test yielded a test statistic of 1043 and an exact p-value of 7.7394 × 10^−35^, indicating a statistically significant difference in the paired classification outcomes. The contingency analysis showed that AFI-ID correctly classified 1,684 instances that were misclassified by HAMC-ID, whereas HAMC-ID correctly classified 1,043 instances that were misclassified by AFI-ID.

A paired bootstrap analysis with 10,000 resamples further supported the observed improvement. The difference between AFI-ID and HAMC-ID was 0.013302 in Macro-F1, with a 95% confidence interval of [0.011168, 0.015426]. The corresponding accuracy difference was 0.012438, with a 95% confidence interval of [0.010478, 0.014437], while the macro-recall difference was 0.013195, with a 95% confidence interval of [0.011083, 0.015325]. All confidence intervals exclude zero.

As shown in Table 24, these results provide statistical support for the observed improvement of AFI-ID over HAMC-ID. Nevertheless, the magnitude of the improvement is relatively modest and should not be interpreted as evidence of universally superior performance. Instead, the results indicate that the proposed hybrid adaptive feature-intelligence mechanism provides a measurable and statistically supported improvement under the controlled experimental setting used in this comparison.

**Table 24:** Five-seed comparison of AFI-ID and HAMC-ID under identical experimental splits.

Model	Accuracy (%)	F1 (%)	Precision (%)	Recall (%)	MCC	Kappa	Log Loss	Training time (s)	Inference/ sample (ms)
AFI-ID	92.70 ± 0.18	94.17 ± 0.15	96.16 ± 0.25	92.26 ± 0.33	0.8455 ± 0.0036	0.8442 ± 0.0037	0.1514 ± 0.0029	18.76 ± 0.16	0.0023 ± 0.0002
HAMC-ID	89.57 ± 0.23	91.60 ± 0.24	94.37 ± 0.47	88.99 ± 0.87	0.7811 ± 0.0028	0.7787 ± 0.0038	0.1889 ± 0.0038	20.84 ± 0.54	0.0023 ± 0.0001

Results are reported as mean ± standard deviation across five random seeds (7, 21, 42, 84, and 123).

## 7 Discussion

### 7.1 Hybrid adaptive feature intelligence

The performance improvements of AFI-ID stem from the complementary integration of statistical relevance and model-based feature importance. Filter-based scoring captures global dependencies between features and class labels, while wrapper-based importance reflects model-specific predictive contributions. By combining these perspectives through weighted fusion, the framework effectively suppresses redundant attributes while preserving discriminative information.

The observed stability across different random seeds indicates that the hybrid ranking process is relatively stable under the evaluated sampling conditions, supporting reproducibility of the reported results. Unlike unsupervised dimensionality reduction methods that prioritize variance, the supervised hybrid approach emphasizes class-discriminative features. This produces a compact feature representation while retaining information that is discriminative under the evaluated data distributions.

### 7.2 Attention-based stacking

Traditional stacking methods rely on static meta-classifiers that assume equal contribution from all base learners. In contrast, AFI-ID utilizes structured meta-features, including prediction confidence, log-probability representations, and entropy-based uncertainty. The BiLSTM module models inter-classifier dependencies, while the attention mechanism assigns context-aware importance weights based on contextual relevance.

This attention-guided weighting strategy allows the meta-classifier to emphasize more reliable predictions while reducing the influence of uncertain outputs. The per-class results indicate that attention-based fusion can provide effective discrimination for several attack categories; however, performance remains sensitive to class frequency, particularly for extremely rare categories. Therefore, the contribution of attention-based fusion to minority-class detection is assessed using per-class Precision, Recall, and F1 rather than aggregate performance alone.

### 7.3 CICIDS2017 limitations, distribution shift, and concept drift

The CICIDS2017 results should be interpreted in the context of several dataset-level limitations. The flow-based representation aggregates network activity into individual flows, while flows generated within the same attack scenario or communication session may exhibit temporal and session-level dependencies. Because explicit session identifiers are not consistently available in the public flow representation, the 60:20:20 stratified partition cannot guarantee complete session-level independence. Consequently, correlated flows may occur across partitions and may lead to somewhat optimistic estimates of generalization performance. The separation of training, validation, and testing data before SMOTE and model evaluation reduces preprocessing-related leakage, but cannot completely eliminate dataset-level temporal dependence.

CICIDS2017 also exhibits substantial class imbalance and dataset bias. In the evaluated multiclass test partition, several attack categories have very limited support. In particular, Infiltration has only one test instance, while Web Attack–SQL Injection has no test instances. Accordingly, the F1-score of 0 for Infiltration reflects failure on a single test instance, whereas the zero value for Web Attack–SQL Injection is not directly interpretable because no test instance is available. Other low-support categories also show substantially weaker F1-scores than dominant classes. Therefore, the aggregate accuracy of 93.54% and micro-averaged ROC-AUC of 0.9992 should not be interpreted as uniformly strong detection across all attack categories. Macro-F1, Balanced Accuracy, MCC, macro PR-AUC, and per-class metrics provide a more informative assessment of minority-class behavior.

The dataset further represents a predefined set of traffic scenarios, attack types, network configurations, and traffic-generation conditions and therefore may not fully represent contemporary operational network traffic. Changes in application behavior, protocols, traffic volumes, attack mechanisms, and class frequencies may produce distribution shift between training and future deployment data. Concept drift may additionally occur when the relationship between traffic characteristics and intrusion classes changes over time. Since AFI-ID uses a supervised static learning procedure and does not explicitly implement continual or online adaptation, the reported results should be interpreted as performance under the evaluated benchmark distributions rather than as evidence of robustness to future or previously unseen traffic conditions. Temporally separated, cross-environment, and continual-learning evaluations would provide stronger evidence of resilience to distribution shift and concept drift.

### 7.4 Computational complexity and empirical efficiency

To complement the theoretical analysis, empirical runtime measurements were obtained under identical experimental conditions for AFI-ID and HAMC-ID. The resulting computational measurements are presented in Table 25. AFI-ID required 24.202 s for training and 0.133841 s for inference, corresponding to 0.002597 ms per test sample, whereas HAMC-ID required 27.773 s, 0.134234 s, and 0.002605 ms per sample, respectively. Thus, in this controlled comparison, AFI-ID required less training time while exhibiting essentially comparable inference latency. These measurements provide empirical evidence of the computational behavior of AFI-ID under the evaluated configuration, but they should not be interpreted as hardware-independent or deployment-scale throughput benchmarks.

**Table 25:** Empirical computational comparison of AFI-ID and HAMC-ID under identical experimental conditions.

Model	Training time (s)	Inference time (s)	Inference/ sample (ms)
AFI-ID	24.202	0.133841	0.002597
HAMC-ID	27.773	0.134234	0.002605

Runtime measurements were obtained under identical experimental conditions. The reported values are hardware- and implementation-dependent and should not be interpreted as universal or deployment-scale benchmarks.

The reduced feature representation decreases the number of input attributes processed by the downstream classifiers; the resulting computational effect depends on the classifier and implementation. The measured runtime should be interpreted within the stated experimental environment rather than generalized to arbitrary production systems.

### 7.5 Deployment considerations

AFI-ID is designed for an offline-trained supervised intrusion detection setting in which feature selection and model training are performed before deployment and the trained model is subsequently used for flow-level classification. The controlled runtime measurements show that AFI-ID has low measured inference latency under the experimental configuration used in this study. However, these measurements do not establish production-scale throughput, memory consumption, energy efficiency, or performance across different hardware platforms.

The reduced feature representation and fixed number of base learners provide a computationally bounded inference architecture, but the complete deployment cost also includes preprocessing, feature extraction, base-learner prediction, meta-feature construction, and attention-based meta-classification. Consequently, the present results should be interpreted as benchmark-level evidence of computational feasibility rather than validation of a production deployment.

Deployment in evolving network environments also presents additional challenges. AFI-ID is supervised and therefore depends on labeled examples of the traffic distributions represented during training. Changes in network applications, traffic characteristics, attack mechanisms, and class frequencies may produce distribution shift between training and operational data. More substantial changes in the relationship between traffic features and attack labels may result in concept drift. AFI-ID does not explicitly implement continual learning or online adaptation to address these changes. Future work should therefore evaluate the framework using temporally separated and cross-environment traffic and investigate continual-learning, online-adaptation, or complementary anomaly-detection mechanisms.

The severe class imbalance observed in CICIDS2017 further limits the interpretation of deployment readiness. Extremely rare attack categories may have insufficient representation for reliable supervised learning, and benchmark performance on such categories cannot be assumed to translate directly to operational detection capability. Accordingly, the present study demonstrates the effectiveness of AFI-ID under the evaluated benchmark distributions but does not claim validated real-world deployment performance or guaranteed detection of previously unseen attacks.

## 8 Conclusion

This study presented AFI-ID, an intrusion detection framework that integrates hybrid adaptive feature intelligence with attention-guided stacking for intrusion detection in high-dimensional and class-imbalanced network traffic. The framework combines statistical relevance measures, including Mutual Information and Chi-square, with model-based feature importance from XGBoost and Extra Trees to construct a compact feature representation. At the ensemble level, Logistic Regression, Extra Trees, and XGBoost generate structured meta-features comprising log-probability representations, prediction confidence, and entropy-based uncertainty, which are subsequently processed by an attention-based BiLSTM meta-classifier.

The experimental results demonstrate that the proposed feature-selection and attention-based fusion strategy provides strong aggregate performance under the evaluated benchmark conditions. AFI-ID achieved 93.31% accuracy in binary classification on UNSW-NB15 and 98.33% on CICIDS2017. The repeated binary experiments across five random seeds further indicated stable performance under the evaluated training conditions. The controlled comparison with HAMC-ID also showed an improvement in aggregate performance, with statistical evidence from McNemar's exact test and paired bootstrap analysis supporting the observed difference under the evaluated test partition.

The component-wise and ablation analyses further indicate that both the hybrid feature-intelligence mechanism and the attention-based stacking architecture contribute to the overall performance of AFI-ID. However, the CICIDS2017 multiclass evaluation demonstrates that aggregate performance does not translate uniformly across all attack categories. Extremely rare classes with very limited test support remain difficult to detect, highlighting the limitations of benchmark-level evaluation under severe class imbalance.

Several limitations should therefore be considered when interpreting the results. The evaluation relies on publicly available benchmark datasets and supervised learning, and the CICIDS2017 flow representation does not provide sufficient session information to guarantee complete session-level independence between partitions. Consequently, the reported CICIDS2017 results may be influenced by temporal or flow-level dependencies and should not be interpreted as evidence of robustness to future distribution shifts or concept drift. In addition, the current AFI-ID framework performs offline supervised learning and does not explicitly implement continual or online adaptation.

## 9 Future work

Future research will prioritize leakage-controlled evaluation using temporally separated and, where feasible, session- or environment-aware data partitions to obtain more rigorous estimates of cross-time and cross-environment generalization. A second priority is rare-class detection, including the investigation of imbalance-aware learning, rare-class augmentation, and class-sensitive optimization strategies for attack categories with extremely limited representation. A third priority is concept-drift adaptation, through continual-learning and online-learning mechanisms capable of updating the detection model as network traffic and attack characteristics evolve.

Further work will also evaluate AFI-ID on additional heterogeneous intrusion-detection datasets and across different network environments to assess transferability beyond the benchmark distributions considered in this study. Lightweight model variants and real-time evaluation will be investigated subsequently, with particular attention to end-to-end preprocessing and inference costs. These extensions are intended to determine whether the observed benchmark-level performance and computational characteristics can be maintained under more realistic and evolving network conditions.

## Data Availability

The original contributions presented in the study are included in the article/supplementary material, further inquiries can be directed to the corresponding author.
